# Disruption of ATP Synthase Spatiotemporal Organization, Ca^2+^ Dynamics, and Contractile Function in Senescent Cardiomyocytes

**DOI:** 10.1111/acel.70388

**Published:** 2026-01-28

**Authors:** Silke Morris, Nico Marx, Gonzalo Barrientos, Isidora Molina‐Riquelme, Frank Schmelter, Hugo E. Verdejo, Stefan Peischard, Guiscard Seebohm, Verónica Eisner, Karin B. Busch

**Affiliations:** ^1^ Institute of Integrative Cell Biology and Physiology, Faculty of Biology, University of Muenster Muenster Germany; ^2^ School of Biological Sciences, Pontificia Universidad Católica de Chile Santiago de Chile Chile; ^3^ Departamento de Enfermedades Cardiovasculares, Facultad de Medicina Pontificia Universidad Católica de Chile Santiago de Chile Chile; ^4^ Department of Cardiovascular Medicine, Institute for Genetics of Heart Diseases (IfGH) University Hospital Münster Münster Germany

**Keywords:** ATP hydrolysis, ATP synthase organization, calcium dynamics, cardiomyocytes, contractility, cristae architecture, human induced pluripotent stem cells, mitochondrial permeability transition pore (mPTP), senescence, single molecule dynamics

## Abstract

Heart disease is the leading cause of death in the elderly population. Age‐related heart failure is frequently associated with energy deficits in cardiomyocytes. These cells rely on their abundant, cristae‐rich mitochondria for ATP production. ATP synthase, localized along the cristae rims, is central to this process. It is presumed that its function is tightly bound to its spatial organization, but details remain unclear. Here, we explored the spatiotemporal organization of ATP synthase in senescent human iPSC‐derived CM in conjunction with its functions. We found changes in the stoichiometry of F_1_ and F_O_ subunits in senescent CM. The ratio of F_O_‐SU c to F_1_‐SU β increased. The oligomeric organization of the complex was weakened. Using single‐molecule localization and tracking microscopy, we observed an increased enzyme mobility within cristae that displayed increased fenestrations. This coincided with decreased mitochondrial ATP level, increased ATP hydrolysis capacity, and a moderate increase in mitochondrial transition pore opening. Disturbed ATP production was correlated with dysregulated calcium dynamics, characterized by heightened spikes and slower cytosolic clearance. Consequently, senescent cardiomyocytes exhibited irregular autonomous and paced beating patterns. These findings indicate that, in senescent cardiomyocytes, functional decline is closely linked to disrupted ATP metabolism, driven by the aberrant organization, dynamics, and activity of ATP synthase within remodeled cristae.

## Graphical abstract

Enhanced ATP hydrolysis, ΔΨm flickering, and destabilized ATP synthase dimers collectively reduce ATP availability in senescent cardiomyocytes. This energy deficit is associated with disrupted Ca^2^⁺ dynamics and contractility.

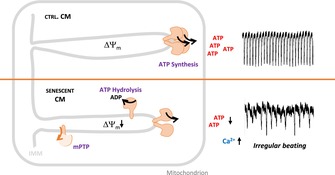

## 1 Introduction

Cardiovascular diseases (CVDs) are tightly coupled to aging and are the leading cause of death in the elderly population, accounting for almost 40% of all age‐related ailments (Fajemiroye 2018). The process of aging and the onset of age‐related CVDs are strongly linked to reduced energy metabolism and decreased mitochondrial function (Abdellatif et al. 2023). In a healthy human heart, more than 90% of ATP is produced by mitochondria through oxidative phosphorylation (OXPHOS), a process that relies on the electron transport chain (ETC; Complexes I–IV) and ATP synthase (Complex V) (Zuurbier et al. 2020). In the rat heart, the function of both ETC and ATP synthase declines with age (Capozza et al. 1994). The OXPHOS complexes are located within cristae, which are folds of the inner mitochondrial membrane (IMM) that increase surface area and compartmentalize mitochondrial processes (Gilkerson et al. 2003). Cristae are connected to the inner boundary membrane (IBM) of the IMM via narrow openings called cristae junctions, which can restrict the diffusion of membrane proteins (Wilkens et al. 2013). The architecture of cristae is crucial for the organization and optimal function of OXPHOS complexes (Stephan et al. 2019; Cogliati 2013) and, vice versa, mitochondrial function can influence cristae structure (Cogliati et al. 2016). Cardiomyocytes (CM) are rich in mitochondria with densely packed cristae (Mannella 2020), and aging is associated with changes in both cristae architecture and CM morphology (Bou‐Teen et al. 2022; Wang et al. 2020).

Obtaining primary aged human cardiomyocytes for physiological characterization is essentially impossible; therefore, hiPSC‐derived cardiomyocytes are a feasible model system, where senescence can be induced following established protocols. We here applied doxorubicin to induce senescence in a controlled way similar as we recently established (Morris et al. 2023). In our previous work, we confirmed the senescence of single hiPSC‐CMs via canonical senescence markers (p16, p21, SA‐β‐gal, γH2AX foci, mitochondrial ROS) (Morris et al. 2023). Yet, there are some limitations, for example, hiPSC‐CMs display premature phenotypes lacking a T‐tubule network (Lundy et al. 2013; Gherghiceanu et al. 2011), which could make the excitation–contraction coupling less efficient (Yang et al. 2014). However, a recent publication showed that the cell capacitance‐to‐volume ratio and cytoplasmic calcium dynamics in hiPSC‐derived cardiomyocytes (CMs) were similar to those in rabbit CMs (Hwang 2015). Differentiation of monolayers of CM on Matrigel has also improved the homogeneity of the CM culture and yielded relatively pure samples (Hwang 2015). Based on these improvements, hiPSC‐derived cardiomyocytes were used to study aging/senescence of cardiomyocytes (Lazzarini 2022; Emanet et al. 2025).

Previously, we characterized hiPSC‐CM and primary rat neonatal ventricular CM (NVCM) after induction of senescence using mild doxorubicin treatment (Morris et al. 2023) and found that both models exhibited compromised IMM ultrastructure and altered cristae morphology. The shape of cristae is determined by several proteins (Frezza 2006; Anand et al. 2021), including ATP synthase, whose dimers and oligomers at cristae tips help to stabilize the cristae structure (Davies 2012; Paumard et al. 2002).

The spatiotemporal organization of ATP synthase is closely linked to its function (Salewskij et al. 2020). When ETC activity is compromised, as occurs during aging, ATP synthase can operate in reverse, hydrolyzing ATP to pump protons into the intracristal space (ICS) (Di Lisa 1995; Rieger et al. 2021). This reverse activity helps maintain the electrochemical membrane potential (Δ_m_) when ATP production by OXPHOS is unfavorable (Garlid and Paucek 2003; Maguire 2006). Previously, we showed that increased ATPase activity is associated with higher mobility of ATP synthase, likely due to a reduction in its dimeric and oligomeric forms (Salewskij et al. 2020). Consistently, defective dimerization of ATP synthase has been observed in aged CM in mouse heart (Bou‐Teen et al. 2022).

Calcium (Ca^2+^) plays a central role in CM. Transient increases in cytosolic Ca^2+^ trigger actin–myosin contraction, while mitochondrial Ca^2+^ uptake stimulates ATP production (Denton and McCormack 1980). The ATP generated is essential for both contraction and the restoration of Ca^2+^ homeostasis between beats, which is mediated by the Na^+^/Ca^2+^ exchanger (NCX), the Na^+^/K^+^‐ATPase, the sarcoplasmic reticulum Ca^2+^‐ATPase (SERCA), and the plasma membrane Ca^2+^‐ATPase, all of which work together to finely regulate cytosolic Ca^2+^ levels (Ng et al. 2023).

Here, we asked how the spatiotemporal organization and function of ATP synthase in senescent CM are associated with energy metabolism, Ca^2+^ oscillations, and contractility. We observed decreased mitochondrial ATP levels in senescent CM, along with a loss of F_O_ subunit e and an increase in F_O_ subunit c relative to F_1_ subunit β in ATP synthase, indicating a reorganization of the complex. Consistently, we found increased ATP synthase mobility, enhanced ATPase activity, and alterations in mitochondrial ultrastructure, ultimately resulting in reduced mitochondrial ATP. Additionally, Ca^2+^ oscillations in senescent CM were irregular, leading to abnormal contractile activity. Together, our findings show that mitochondrial ATP deficiency is associated with impaired Ca^2+^ clearance in beating CM and increased susceptibility to mitochondrial transition pore opening (mPTP), which may establish a vicious cycle of disrupted energy homeostasis and declining cardiac function.

## 2 Experimental Procedures

### 2.1 Cell Culture and Differentiation of hiPSC

All cells were routinely cultured in a humidified atmosphere of 5% CO_2_ and 37°C. Senday Foreskin 1 (SFS.1) cells were cultured in FTDA Medium (Frank et al. 2012) on plates coated with Matrigel (Corning, #354263) with daily medium exchange. Cells were passaged when they reached 100% confluency under the presence of Rock‐inhibitor Y‐27632 (R&D Systems, #1254/10). Differentiation into CM (hiPSC‐CM) was carried out according to Peischard (2017). Briefly, cells were seeded at high density of 500,000 cells per well of a 24‐well plate on day 0 of differentiation in differentiation medium (Rao 2016). Concentrations of BMP‐4 were batch dependent and were determined in a first test differentiation. Medium was exchanged daily with fresh TS‐ASC medium (Rao 2016) for 7–10 days until autonomous beating was observed. To ensure differentiation into CM, the WNT pathway was inhibited by adding C59 on Days 2 and 3 of differentiation. After differentiation, cells were frozen in liquid nitrogen. For experiments, hiPSC‐CM were thawed and seeded out on plastic coated with 0.2% gelatin (Sigma Aldrich, #G1393) or glass coated with 0.2% gelatin and FBS (PAN Biotech, #P30‐3031) and cultured for at least 2 weeks prior to experiments.

For ventricular differentiation, beating cardiomyocytes were transferred after 8 days to Matrigel‐coated plates with a layer of gelatine. Cells were then cultured in KO‐THAI medium (KO‐DMEM with PSG, ITS, 0.2% HSA, 250 μM phospho‐ascorbate, 0.008% thioglycerol) for an additional 28 days to develop a more mature ventricular phenotype.

### 2.2 Cardiac Myocyte Isolation and Culture

CM were isolated from the hearts of neonatal Sprague–Dawley rats as described previously (Frank et al. 2012). Rats were bred in the Bioterio of the Pontificia Universidad Católica de Chile. All studies followed the Guide for the Care and Use of Laboratory Animals published by the US National Institutes of Health (NIH Publication No. 85‐23, revised 1996) and were approved by the Institutional Ethics Review Committee. Briefly, ventricles were dissected, pooled, and myocytes dissociated in a solution of collagenase (Worthington #LS004176) and pancreatin (Sigma Aldrich #8049‐47‐6). After enzymatic dissociation, the cells were selectively enriched for cardiac myocytes (NVCMs) by being plated in F‐10 Ham medium (Gibco #11550–043) supplemented with 10% (v/v) horse serum (Gibco #16050‐114), 5% (v/v) fetal bovine serum (Gibco #16000‐044), penicillin and streptomycin (100 units/mL; Thermo Scientific #15140122). The NVCMs were plated on 2% (w/v) gelatin‐coated glass coverslips. Finally, the cells were kept at 37°C in a humid atmosphere of 5% CO_2_. 5‐bromo‐2‐deoxyuridine (Sigma Aldrich #59‐14‐3) was added to prevent the proliferation of remaining fibroblasts.

### 2.3 Induction of Senescence

Senescence was induced by treatment with 50 nM doxorubicin (Doxo, Sigma Aldrich, #D1515) for 96 h according to Morris et al. (2023).

### 2.4 Generation of a Stable Cell Line

For generation of an SFS.1 cell line stably expressing the subunit gamma (SU γ) of the ATP synthase fused to the HaLo tag (SU γ‐HaLo), a triple vector system was used. The main vector was a well‐established stem‐cell Tet‐On transfection vector, KAO717‐pPB‐hCMV1‐IRES‐Venus (A) (Peischard et al. 2020; Estarás 2017; Quaranta et al. 2018), in which we inserted the gene sequence for SU γ‐HaLo (Salewskij et al. 2020; Appelhans 2012). We did this by amplifying the sequence for SU γ‐HaLo from the original vector using primers with overhanging ends containing restriction sites for *XhoI* and *NotI* (highlighted in bold), which allowed cloning of the sequence into KAO717‐pPB‐hCMV1‐IRES‐Venus (B) via the same restriction sites.

Fwd: 5′‐**CTCGAG**ATGTTCTCTCGCGCGGGTGTCG‐3′.

Rev.: 5′‐**GCGGCCGC** ACCGGAAATCTCCAGAGTAGAC‐3′.

Expression of SU γ‐HaLo was hence controlled by the CMV_min_ promoter. The Venus marker that was encoded on the transfection vector allowed for easy detection of positive clones and was controlled by an IRES2 sequence. The second vector was KA0637‐pPgCAG‐rtTAM2‐IN. This vector contained a G418 resistance gene for selection of positive clones, as well as the rTA transactivator sequence that allows doxycycline‐inducible expression of SU γ‐HaLo (Figure S8A). The last vector of the trio was PB200PA‐1 (C). This encoded the PiggyBac transposase that enabled stable insertion into the genome of transfected hiPS cells.

We transfected SFS.1 cells with Lipofectamine 3000 (Thermo Fisher Scientific, #L3000‐001) according to the manufacturer's instructions using the three plasmids at a ratio of A:B:C = 15:3:1. Medium was changed after 8 h. The optimal concentration of G418 was previously determined by generation of a kill curve. We started selection with 50 μg ml^−1^ G418 (Sigma Aldrich, #A1720) 24 h after transfection, then increased this to 150 μg ml^−1^ after 48 h and finally to 200 μg ml^−1^ after 120 h. Selection was considered complete when no more cells were viable in the non‐transfected control, which was 12 days post‐transfection. Cells were then seeded in a dilution series onto a 96‐well plate, which led to monoclonal cultures in some wells. Selection pressure was reduced to 20 μg ml^−1^ for another 5 days before G418 was not added to the culture medium anymore. Monoclonal cultures were expanded and expression was induced with 0.25 μg ml^−1^ doxycycline. Heterogenous expression of Venus (green) and SUγ‐HaLo (labeled with HTL‐TMR) was confirmed via confocal microscopy. Clones that did not express the two constructs were discarded. Subsequently, a test differentiation into CM was carried out as described above.

Before experiments, expression of SUγ‐HaLo was induced by adding 0.25 μg ml^−1^ doxycycline for 96 h.

### 2.5 Quantitative PCR

Total RNA was extracted from differentiated hiPSC‐CMs using the Monarch Total RNA Miniprep Kit (NEB #T2010), and equal amounts of RNA per sample were transcribed into cDNA using the GoScript Reverse Transcription Kit (Promega #A5000). Quantitative PCR (qPCR) was carried out on a StepOnePlus Real Time PCR System (Thermo Scientific) using the PowerUP SYBR Green Master Mix (Applied Biosystems #A25741), between 60 and 80 ng of cDNA and 0.1 nM of each forward and reverse primer per reaction. The PCR program consisted of an initial denaturation step at 95°C for 10 min followed by 40 cycles of 95°C for 15 s and 60°C for 60 s. A melting curve was generated after the run (60°C–95°C at 0.8°C increase per minute). GAPDH served as an endogenous control. Primers were synthesized by Eurofins Genomics (Table 1).

**TABLE 1:** Primers used for qRT‐PCR.

Target	Forward sequence 5′ – 3′	Reverse sequence 5′ – 3
GAPDH	CTG GTA AAG TGG ATA TTG TTG CCA T	TGG AAT CAT ATT GGA ACA TGT AAA CC
NDUFA9	AGC TTC ATC ATG CCC TCA TGC C	TCT CGC GTC CCA TTC CAG AAA C
NDUFV1	TGT GTG AGA CGG TGC TGA TGG A	CGA TGG CTT TCA CGA TGT CCG T
SDHA	GCA AAA TCA TGC TGC CGT GTT C	ATC CGC ACC TTG TAG TCT TCC C
UQCRC3	ATG CTA AAG GAG ATG CCA CTG C	GCC AAC CAT CCA GTT CTT CCT C
Cox8a	AAG ATC CAT TCG TTG CCG C	TAG GTC TCC AGG TGT GAC AG
ATP5F1A	CCA AAC CAG GGC TAT GAA GCA G	CAC CCG CAT AGA TAA CAG CCA C
ATP5F1B	GAA GAC AAG TTG ACC GTG TCC C	TCA CGA TGA ATG CTC TTC AGC C

### 2.6 Western Blotting

Cells were lysed in RIPA Buffer (Thermo Scientific, #89901) with added protease inhibitor (Thermo Scientific, #78430). Protein quantification in lysates was performed by modified Bradford assay (Roti Nanoquant, Roth, #K880). Equal protein amounts were supplemented with 4× SDS‐PAGE sample buffer and subsequently loaded on a 10% (w/v) polyacrylamide gel. After electrophoresis, gels were transferred onto a PVDF membrane. Membranes were blocked with 10% milk, and immunoblotting was performed. Primary antibody was the OXPHOS rodent WB antibody cocktail (abcam, #ab110413); secondary antibody was the goat anti‐mouse antibody (Jackson ImmunoResearch, #115–035‐068). Visualization of bands was enabled by chemiluminescence (SuperSignalTM West PICO PLUS, Thermo Scientific, #34580) and membranes were stained with Coomassie blue for signal normalization. To determine ATP synthase composition, subunits e, IF1, β, and c were immunoblotted (Table 2). Caspase 3 cleavage was tested with an antibody that detects cleaved Caspase 3 (MedChem Express, #HY P80623, rabbit monoclonal).

**TABLE 2:** List of antibodies used for immune detection of ATP synthase subunits and mitochondrial complexes.

Antibody	Provider	Ordering number	Specification
ATP synthase C [EPR13907] (SUc)	Abcam	ab181243	Rabbit monoclonal
ATPB (SUβ = beta]	Abcam (ab170947)	ab170947	Rabbit monoclonal
ATPIF1	Cell Signaling (D6P1Q)	13268S	Rabbit monoclonal
ATP5I (SUe)	Abcam (ab122241)	ab122241	Rabbit polyclonal
Tom20	Proteintech (11802–1‐AP)	11,802–1‐AP	Rabbit polyclonal

### 2.7 Mitochondria Isolation and Blue Native Gel Electrophoresis

Blue Native gel electrophoresis (BN‐PAGE) was conducted according to Wittig, Braun, and Schägger (2006). SUγ‐HaLo SFS.1 and NVCM were grown in a T75 culture flask to confluency. After harvesting, the cell pellet was resuspended in ice cold mitochondrial isolation buffer consisting of 300 mM sucrose, 5 mM TES, and 200 μM ethylene glycol tetraacetic acid (EGTA), pH 7.2. Cells were then lysed mechanically by slowly passing the cell suspension six times through a cell homogenizer (Isobiotec) using a 6 μm clearance tungsten carbide ball and glass syringes (Hamilton). The obtained lysate was subjected to centrifugation at 4°C, 800 × *g* for 10 min 3 times to remove nuclei and cell debris. The mitochondria‐containing supernatant was subsequently centrifuged at 4°C, 9000 × *g* for 15 min. The resulting pellet contained mitochondria and was resuspended in mitochondrial isolation buffer. Protein concentration was quantified by Bradford assay (ROTI Nanoquant, Roth, #K880.3). 50 μg protein was subjected to another round of centrifugation at 4°C, 9000 × *g* for 10 min and the resulting pellet was resuspended in blue native sample buffer (Wittig, Braun, and Schägger 2006) supplied with 4% digitonin (Sigma Aldrich, #D5628). Samples were separated on a 3%–12% polyacrylamide gradient gel (Serva, #43250) alongside a molecular weight marker (NativeMark, Thermo Fisher Scientific, #LC0725). After electrophoresis, the gel was transferred to a PVDF membrane and western blotting was performed using an anti‐Halo primary antibody (Promega, #G9211) and a goat anti‐mouse secondary antibody (Jackson ImmunoResearch, #115‐035‐068). For visualization of Complex V, anti‐ATPB (subunit β of the ATP synthase, abcam, cat# ab170947) was used as a primary antibody and goat anti‐rabbit (Jackson ImmunoResearch, cat# 111‐035‐045) as secondary antibody. Bands were visualized by chemiluminescence (Super Signal West PICO PLUS, Thermo Fisher Scientific, #34580).

### 2.8 Live Cell Imaging

Fluorescence microscopy was carried out on a confocal laser scanning microscope (Leica TCS SP8 SMD) equipped with a 63× water objective (N.A. 1.2) and a tunable white light laser. HyD's with GaASP photocathodes were used as detectors. Measurements were performed in a humidified chamber at 37°C and 5% CO_2_. Membrane potential was determined by staining the cells with 7 nM tetramethylrhodamine ethyl ester perchlorate (TMRE) (Thermo Scientific, #T669) and 100 nM MitoTracker Green (MTG, Thermo Scientific, #M7514) for 30 min and subsequent imaging. Before imaging, MTG was washed out, while TMRE was kept during imaging. Image analysis was carried out in ImageJ by measuring the fluorescence intensity of single cells in both channels. The TMRE signal (excitation: 561 nm, emission: 570–695 nm) was then normalized pixelwise to the MTG signal (excitation: 490 nm, emission: 495–547 nm). In a separate experiment, loss of mitochondrial membrane potential was induced by addition of 10 μM FCCP, and time lapses were recorded at an acquisition rate of 1 frame per 3 s.

ATP levels were determined by staining with 10 μM of the fluorogenic dye BioTracker ATP‐Red (Sigma Aldrich, #SCT045). Before measurements, cells were stained with MTG (100 nM for 30 min). Image analysis was carried out in ImageJ by measuring the fluorescence intensity of single cells in both channels and normalizing the ATP‐Red intensity to the MTG intensity.

### 2.9 Immunofluorescence Staining

For immunofluorescence staining, cells were cultured on cover glasses, and washing with PBS was carried out after each step. Prior to staining, cells were fixed with 4% PFA for 20 min at room temperature. Residual PFA activity was quenched by incubation in 0.1 M Glycine for 10 min. Cover glasses were then incubated in 0.1% Triton X‐100 in PBS for 4 min to allow permeabilization of the cells and subsequently blocked in 5% normal goat serum in PBS for 30 min. Staining was then carried out by incubating the cover glasses with the primary antibody anti‐α‐actinin (Sigma Aldrich #A7811) diluted 1:400 in blocking buffer for 60 min and with the secondary antibody pAb to RbIgG, tagged with Alexa Fluor 555 (abcam, #150078) diluted 1:400 for 60 min. Coverslips were then mounted onto microscope slides and left to dry overnight before imaging (excitation: 555 nm, emission: 565–610 nm).

### 2.10 Calcein/Co^2+^ Quenching Assay

The green fluorescent dye calcein enters the cell and mitochondria. MitoTrackerDeepRed (MTDR) stains mitochondria. When Co^2+^ is present, it quenches calcein in the cytosol and in mitochondria, if the mPTP is open (Petronilli et al. 1999). Thus, the MTDR/calcein signal reports mPTP opening. Cells are washed in modified HBSS solution containing 10 mM HEPES, 10 μM GlutaMAX, and 100 μM NV118. Calcein (2 μM) and Co^2+^ (Cobalt (II) chloride hexahydrate, 1 mM) are added together with 200 nM MTDR for 15 min at 37°C and 5% CO_2_. Then, cells are washed and imaged. Excitation calcein: 495 nm, emission: 505–636 nm; excitation MTDR: 641 nm, emission: 651–790 nm using a cLSM (Leica SP8) equipped with a white light laser, two HyD detectors and a 63× water objective (1.2 NA).

### 2.11 TMRE Flickering Assay

Cells were stained with MTG (100 nM) and TMRE (12.5 nm) for 30 min in culture medium without phenol red (imaging medium). Before imaging, MTG was washed out, while TMRE was kept during imaging. For testing Cyclosporin A (CsA) effects, cells were pre‐incubated with 2 μM CsA for 30 min. Measurements were performed in the presence of CsA and TMRE after pre‐staining with MTG. Excitation TMRE: 561 nm, MTG: 488 nm. 10–15 × 10–15 μm regions of interest (ROIs) were randomly chosen for recording. Pixel size was 50–80 nm. 100 frames were recorded within a total imaging time of 1 min 20 s. Flickering events were manually counted per ROI. A flickering event was positive if a sudden drop in TMRE appeared which recovered during the next frames as described in (Sambri 2020). Microscope: cLSM (Leica SP8).

### 2.12 ATP Hydrolysis Assay

**FIGURE 1:**
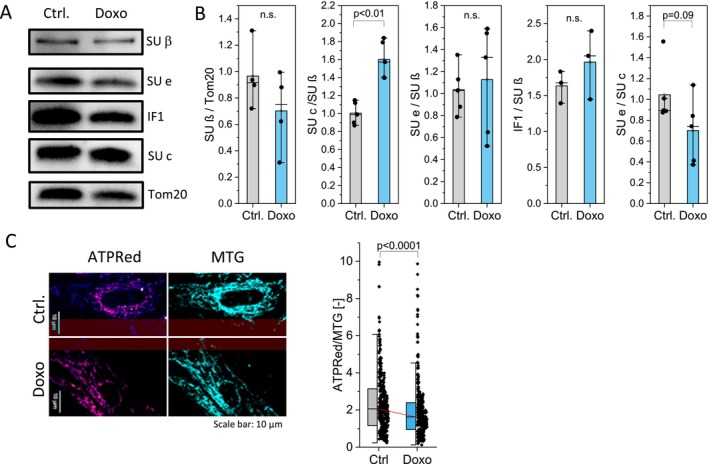
Remodeled ATP synthase composition is linked to decreased ATP levels in senescent cardiac cells. hiPSC‐CM were treated with 50 nM doxorubicin for 4 days (Doxo) to induce senescence or left untreated (Ctrl). (A) Protein amount of subunits β (ATP5B), e (ATP5I), IF1 (ATPIF1), and c (ATP5MC) in control and senescent hiPSC‐CM cells. Tom20 is used as a loading control. (B) Relative abundance of ATP synthase subunit proteins. hiPSC‐CM treatments: *n* = 5 (SU e/SU β), (SU c/SU β), *n* = 3 (IF1/SUβ) and *n* = 4 (SUβ/Actin). Mann–Whitney test. (C) In hiPSC‐CM, we determined mitochondrial ATP levels by ATP‐Red1, normalization to MitoTrackerGreen for mitochondrial mass (MTG). ATP‐Red1 is shown as hot color‐coded. Statistical test: Mann–Whitney test, *n* = 3 replicates, *N* = 336 single cells. Single points indicate measurements of single cells.

To determine ATP hydrolysis by complex V, the HyFS (Hydrolysis in frozen samples) assay was performed using an extracellular flux analyzer (Seahorse XF96) as previously described in (Fernandez‐Del‐Rio et al. 2023). Cell pellets harvested from a 6 well plate were resuspended in 100 μL of MAS buffer and put through four freeze–thaw cycles alternating between −80°C (freezer) and +37°C (water bath). Protein concentrations were determined, and 20 μg of cell homogenate was loaded into a well of a Seahorse XF96 microplate in 20 μL of MAS. The plate was centrifuged at 2000 *g* for 10 min at 4°C (no brake), and then 130 μL of MAS containing 5 mM succinate/2 μM rotenone and 100 μg/mL of cytochrome c was added to each well to allow for electron transfer in between OXPHOS complexes resulting in oxygen consumption by Complex IV. Oxygen consumption rates (OCR) and acidification rates (AR) were recorded. Injections were performed as indicated in the figure panels at the following final concentrations: antimycin A (AA, 2 μM), FCCP (1 μM), ATP (20 mM), and oligomycin (5 μM). To achieve maximal ATP concentration, two ports of ATP/FCCP were injected simultaneously. The hydrolytic capacity was quantified by the maximal hydrolysis by CV (AR signal after ATP) – (AR signal after oligomycin) and normalized by the protein contents of CV vs. Tom20, which were determined by immunoblotting of each sample loaded in the assay (Figure 1A).

### 2.13 Tracking and Localization Microscopy

Tracking and Localization Microscopy (TALM) was carried out as previously described (Appelhans and Busch 2017a). Differentiated hiPSC‐CM were treated with doxorubicin as described or left untreated. 96 h prior to the experiment, expression of SU γ‐HaLo was induced using 0.25 μg ml^−1^ doxycycline. One day before the experiment, cells were transferred onto ibidi glass bottom dishes (ibidi, #81156). For imaging, cells were stained with 1 nM Halo Tag Ligand (HTL) tagged with Janelia Fluor 646 (HTL‐JF646, Promega, #GA1110) for 30 min at 37°C. Subsequently, cells were washed three times with PBS, and phenol red‐free medium was added to the cells for imaging. Cells were imaged at room temperature on an Olympus TIRF 4 Line system equipped with a 100× oil immersion TIRF objective (N.A. 1.49) and a diode pumped solid‐state laser (excitation 640 nm, 140 mW, emission filter: 600/37 Brightline HC). Imaging was carried out with a highly inclined and laminated optical sheet (HILO) (Tokunaga et al. 2008), which was achieved by decreasing the illumination angle just below the critical angle for total internal reflection (TIR). 5000 frames were recorded at a frame rate of 58 Hz (17 ms per frame) with a back‐illuminated highly sensitive camera (Hamamatsu CMOS Model C14440‐20UP, pixel size 130 nm).

The labelling density was low enough to allow the identification of single molecules. To confirm mitochondrial localization of the signals, a cumulative projection of all frames was generated. Localization and generation of single molecule trajectories were carried out based on algorithms described by Sergé (Sergé 2008). These were implemented in a custom‐made MATLAB code, SLIMfast (provided by C.P. Richter, University of Osnabrück). Briefly, the point spread function (PSF) was experimentally determined by fitting a 2D Gaussian function over the signals. Based on this, single molecules were localized in each frame with an error probability of 10^−7^. Localizations were then connected between frames to form trajectories. Only trajectories that consisted of at least five steps (85 ms) were included in the analysis, and the search radius was limited to be between 13 and 130 nm (0.2 and 1 pixel).

### 2.14 Ca^2+^ Imaging

Cytosolic Ca^2+^ transients were visualized using the fluorescent indicator Fluo‐4‐AM (Fluo‐4; Biomol/ATT Bioquest, ABD‐20551). For reference, cells were co‐stained with Fura‐Red (Fura‐Red; Biomol/ATT Bioquest, ABD‐21048). Both dyes were added at 2 μM final concentration 25 min before measurements. Then, cells were washed and imaged. Calcium transients were recorded over a period of 4 min with a frame rate of 0.74 s at 37°C and 5% CO_2_. Excitation: 488 nm, Fluo‐4 emission: 500–530 nm; Fura‐Red emission: 600–727 nm using a cLSM with white light laser excitation and a 20× dry objective (0.75 NA). Only beating CMs were analyzed by measuring manually traced ROIs of single cells.

The Peak Analyzer tool in Origin Pro was used to fit Calcium traces from the measured Fluo‐4 fluorescent intensities. A baseline was created using the Asymmetric Least Squares (ALS) Smoothing method with an asymmetric factor of 0.01 and a smoothing factor of 5. Local maxima were determined automatically to allow for subsequent fitting of each peak independently.

For peak fitting of control CMs, a gaussian function was used.

(1) $$y=y_{0}+\frac{A}{w\times \sqrt{\frac{\pi }{4\times \ln\left(2\right)}}}\times e^{\left(-4\times \ln\left(2\right)\times \frac{{\left(x-x_{c}\right)}^{2}}{w^{2}}\right)},$$

where y_0_ is the offset (baseline), *A* is the peak area, *w* is the peak width, and x_c_ is the peak center.

Since calcium traces of coxo‐treated CMs show a prolonged decay period in each peak, a user‐defined exponentially‐modified gaussian fitting function was used to increase the accuracy of the fit:

(2) $$y=y_{0}+h\times \frac{\tau }{2}\times e^{\left(\frac{w^{2}}{2\tau^{2}}-\frac{\left(x-x_{c}\right)}{\tau }\right)\times \mathrm{erf}c^{\left(\frac{w}{\sqrt{2\times \tau }}-\frac{\left(x-x_{c}\right)}{\sqrt{2\times w}}\right)}},$$

where *h* is the peak height (its amplitude at the peak center *x*_*c*_), τ is the exponential decay constant and *erfc* () is the complementary error function with *erfc* (x) = 1‐ *erf* (x) calculated via the Gauss error function *erf* (x).

### 2.15 CardioExcyte 96 Analysis

Cardiac‐like cells derived from hiPSCs were plated on 0.1% gelatine‐coated NSP‐96 plates (Nanion Technologies GmbH) at a density of 30,000 cells per well. Cells were seeded in KO‐THAI and 10 μM Y‐27632. After 24 h, Y‐27632 was removed, and 50 nM Doxorubicin was added to a subgroup of seeded cells for senescence induction. Spontaneous contractions were observed by Day 5. The NSP‐96 plates with hiPSC‐derived cardiac cells were placed in the CardioExcyte 96 device (Nanion Technologies GmbH) to monitor cell activity (contraction via baseline impedance) for 2 h, with measurements taken every 15 min for 30 s at 37°C and 5% CO_2_. The cells' spontaneous activity was measured as well as activity under tachypacing at 3 Hz. Data was processed with CardioExcyte Data Control Software (Nanion Technologies GmbH). Peak‐to‐peak time of contraction signals was analyzed to verify changes in contractile behavior of non‐treated and Doxorubicin‐treated cells.

### 2.16 Electron Tomography

Pellets of NVCM were fixed and stained as described previously (Morris et al. 2023). Sections of 200 nm for tomographs were obtained using the ultramicrotome EM UC7, Leica Ultracut R. Single‐tilt image series (±60°, 1° increment) were acquired in the transmission electron microscope TALOS F200C G2 (Thermo Scientific) equipped with a Ceta 16 M CMOS camera, at 200 kV using a 1024 × 1024 detector array at the Unidad de Microscopía Avanzada de la Pontificia Universidad Católica (UMA‐UC). Raw tomograms were drift‐corrected and aligned using Inspect 3D (Thermo Scientific), applying Expectation Maximization algorithm (EM) with 20 iterations.

### 2.17 Statistical Analysis

For statistical analysis, data was tested for normal distribution (Shapiro–Wilk). In case of normal distribution, statistical significance was determined by One‐Way‐ANOVA followed by Tukey's Range Test. For non‐parametric testing, Kruskal‐Wallis ANOVA was used.

## 3 Results

We recently presented a hiPSC‐CM aging model to study the effects of senescence on mitochondrial OXPHOS, dynamics, and architecture. Senescence was induced by exposure to a low dose of doxorubicin (50 nM, 4 days) (Morris et al. 2023). As shown in Figure S1, this treatment resulted in a significant increase in DNA double‐strand breaks and larger nuclei area, typical features for aged cells (Mah et al. 2010; Pathak et al. 2021). We also detected mitochondrial elongation, which was recently reported to be associated with senescence (Mai et al. 2010). We found reduced mitochondrial OXPHOS function in senescent hiPSC‐CM (Morris et al. 2023). In this study, we characterize the functional activities of ATP synthase, forward ATP synthesis, reverse ATP hydrolysis, and involvement in leak metabolism in more detail and relate it to its spatiotemporal organization.

### 3.1 ATP Synthase Composition and Oligomeric Organization Are Changed in Senescent CM

First, we determined whether senescence affects the amount and molecular composition of ATP synthase. ATP synthase is composed of an F_O_ and F_1_ subcomplex. Several subunits of ATP synthase, namely subunits e/g and inhibitor factor 1 (IF1), stabilize the dimerization or oligomerization of the enzyme (Paumard et al. 2002; Wagner et al. 2010; Dominguez‐Zorita et al. 2023; Weissert et al. 2021). Primarily, IF1 is an inhibitor of ATPase function of ATP synthase (Carroll et al. 2024; Gledhill et al. 2007). An excess of subunit c (SU c) was shown to increase the leak of the inner mitochondrial membrane and was associated with the mitochondrial transition pore (Alavian 2014). Subunit e (SU e) is suggested to block the leak through the c‐channel (Gerle 2020; Pinke et al. 2020). Therefore, SU e, IF1, and SU c are key for ATP synthase activity. Their protein levels were determined by immunostaining and related to SU β, the main subunit of the F_1_ subcomplex (Figure 1A). The amount of SU β normalized to Tom20 was not altered in senescent CM as we reported before (Morris et al. 2023), but we made two striking observations: First, the ratio SU c/SU β was increased in senescent CM, resulting in an imbalance of F_O_ core relative to F_1_ (Figure 1B). Second, we found a tendency for a decreased ratio of SU e/SU c. When individual treatments were compared (Figure S2A), the SU e/SU c ratio was always lower in senescent cells, indicating that the subunits of the F_O_ subcomplex might be independently regulated. IF1 showed no significant change when related to SU β. This data—increase of c relative to β and tendentious loss of e relative to c—indicates a re‐organization of ATP synthase which would likely also affect the dimerization/oligomerization assembly. Because the amount of hiPSC‐CM material is limited, we could not perform native PAGE analysis of ATP synthase organization to see if there are free low molecular weight oligomers of c‐subunits or dimer and oligomer weakening. Instead, senescent rat NVCMs were used as an alternative source (Morris et al. 2023). ATP synthase was separated via BN‐PAGE followed by immunostaining for ATP5B. Although we found substantial variability among samples (Figure S2B), our data insinuated a decrease tendency in ATP synthase dimers in senescent cells, as previously reported (Bou‐Teen et al. 2022; Daum et al. 2013). In the same BN‐PAGE, we also found subcomplexes with a molecular weight around 480 kDa. According to He et al. (2018), these could be subcomplexes composed of F_1_‐c_8_ and F_1_, which were present in control and senescent cells.

### 3.2 Mitochondrial ATP Is Reduced in Senescent hiPSC‐CM

We next tested whether mitochondrial ATP levels were changed in senescent cells. Relative ATP levels were determined with the fluorescent probe ATP‐Red1 (Wang et al. 2015) and normalized to mitochondrial mass via MTG staining. We found a significant reduction of mitochondrial ATP levels from 2.06 to 1.63 a.u. in senescent CM (Median, *p* < 0.001) (Figure 1C).

### 3.3 Senescent CM Show a Higher mPTP Opening Susceptibility

Increase in SU c/SU β strongly suggests an increase in mitochondrial leak due to imbalanced stoichiometry (Alavian 2014). This would reduce the proton motive force and thus ATP synthesis efficiency. To assess this, we monitored ΔΨ_m_ over time using the ΔΨ_m_‐sensitive dye Tetramethylrhodamine‐ethylester (TMRE) in the non‐quenching mode (Perry 2011). The specificity of TMRE was tested by recording the effect of the uncoupler FCCP on ΔΨ_m_ (Figure S3).

**FIGURE 2:**
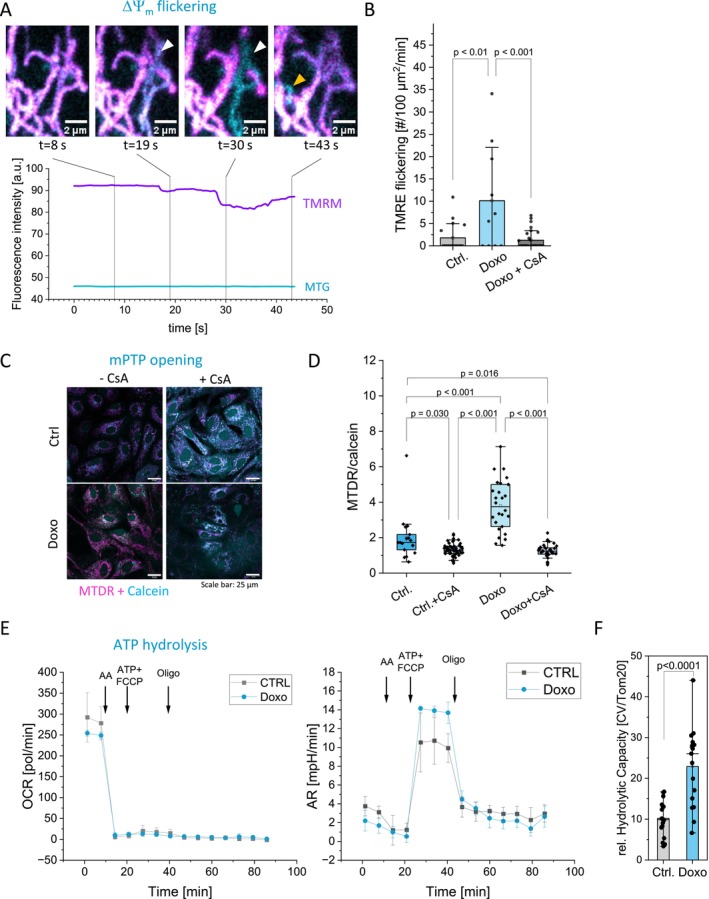
Senescent CM exhibit enhanced mPTP opening, ΔΨm flickering and ATP hydrolysis. (A) ΔΨ_m_ flickering: Example images illustrating a flickering event in a doxorubicin‐treated senescent hiPSC‐CM cell. The TMRE signal (purple) is lost in a spatially and temporally restricted manner due to the transient opening of the mPTP, but recovers by the end of the event at *t* = 43 s. The Mitotracker Green signal (MTG; cyan) remains stable throughout the event. The graph shows the fluorescence intensities of TMRM and Mitotracker Green between 0 and 50 s in the above region. (B) Flickering frequency in control and senescent cardiomyocytes. Cyclosporin A (CsA) reduces flickering. Ctrl. and Doxo: *n* = 5, Doxo + CsA *n* = 3. Statistical test. Dunn‐Sidak. (C) Co^2+^/calcein quenching assay to determine relative leakiness of the IMM. (D) Quantification of mitochondrial leak indicated by loss of calcein fluorescence in mitochondria; ratio of MitotrackerDeepRed (MTDR) and calcein fluorescence in control and senescent CM. *n* = 2. 100 cells (Ctrl.), 61 cells (Doxo). Statistic test: Dunn‐Sidak. See also Figure S3. CsA effect on mPTP opening: *N* = 1, single points indicate mean values of cells. (E) ATP hydrolysis assay measuring oxygen consumption rate (OCR) and acidification rate (AR). (F) Quantification of the hydrolytic capacity of ATP synthase in control and senescent cells normalized on CV/Tom20 protein levels. Statistics: Anova. *n* = 5 treatments.

ΔΨ_m_ was determined via TMRE‐MTG double staining: while TMRE is sensitive to membrane potential, MTG, once taken up, is covalently bound to mitochondrial proteins allows for normalization (Protasoni et al. 2024). We used these double stained cells to record time courses following the TMRE‐MTG signal over time. Transient opening of the mPTP results in transient loss of the TMRE, but not the MTG signal (Protasoni et al. 2024). We observed frequent flickering of the TMRE signal while MTG fluorescence remained stable, indicating transient losses of ΔΨ_m_ (Figure 2A). As indicated by the yellow and white arrowheads, two flickering events are shown here. The mitochondrion marked in yellow does not fully recover by the end of the measurement, meaning that the original TMRE signal strength is not achieved. These transient depolarizations were more frequent in senescent cells compared to controls and were suppressed by Cyclosporin A (CsA), a known mPTP inhibitor (Carraro et al. 2020) (Figure 2B). Our findings are consistent with recent studies reporting increased transient mPTP openings in senescent cells (Bou‐Teen et al. 2022; Protasoni et al. 2024). Additionally, we applied the calcein/Co^2+^ quenching assay to determine leakiness of the IMM for ions (Petronilli et al. 1999). Calcein is a cell‐ and mitochondrial‐permeable dye that is quenched by Co^2+^. Although Co^2+^ can cross the plasma membrane, it does not enter mitochondria unless they become leaky, for example, by mPTP opening (Figure S4). Cells were loaded with calcein and Co^2+^ and mitochondria were stained with MTDR, whose retention within mitochondria is ΔΨ_m_‐independent. MTDR is not quenched by Co^2+^. Therefore, the ratio of MTDR and calcein is an indicator for mPTP opening. We observed a significant higher MTDR/calcein ratio in senescent CM indicating mitochondrial Co^2+^ influx over time (Figure 2C,D). The mPTP antagonist CsA blocked the mitochondrial calcein quenching confirming the specificity of the assay (Figure 2D). The Co^2+^ quenching of mitochondrial calcein can be explained by transient opening and influx of few Co^2+^ ions and does not require permanent opening of the mPTP. Doxorubicin per se did not affect mPTP opening in isolated mitochondria as we ruled out by a swelling assay, which is a typical measure for mPTP opening (Carraro et al. 2020; Petronilli et al. 1993; Figure S5). Furthermore, we tested, whether increased frequency of mPTP would provoke apoptosis. Therefore, we tested for pro‐caspase 3 cleavage. We found no cleaved caspase 3 suggesting that transient mPTP openings, even if more frequent, do not evoke apoptosis under unchallenged conditions (Figure S6). In sum, we found intermittent loss of ΔΨ_m_ that is associated to an overall susceptibility to mPTP opening in senescent CM but without imminent activation of apoptosis.

### 3.4 ATP Hydrolysis Capacity Is Enhanced in Senescent Cardiomyocytes

We next asked whether re‐organization of ATP synthase and decreased ΔΨ_m_ could be a trigger for ATP hydrolysis. To check ATP hydrolysis capacity, we harvested control and senescent cells and subjected them to multiple cycles of thawing and freezing. This is a mild procedure for permeabilization but maintains coupled mitochondria (Fernandez‐Del‐Rio et al. 2023). The cells were then provided with substrate to stimulate the respiratory chain. To check for equal loading, samples of the seeded cells were immune‐stained for the outer mitochondrial membrane protein Tom20. OCR and AR were measured with an automatic flux analyzer (Seahorse XF96/Agilent). To block respiration, antimycin A was added, and mitochondria were uncoupled by addition of FCCP in combination with high concentrations of ATP to stimulate reverse ATP synthase function. OCR and AR were determined before and after addition of oligomycin which allows us to calculate the ATP hydrolysis capacity of ATP synthase (Fernandez‐Del‐Rio et al. 2023; Figure 2E). Indeed, we found a significant increase of the ATP hydrolysis capacity in senescent cells (Figure 2F).

### 3.5 Senescence Is Associated With Increased ATP Synthase Mobility

Given the relative changes of subunit composition and increase in ATP ase function of ATP synthase in senescent cells, we investigated how these changes affect its localization and mobility within the IMM. We hypothesized that increased levels of monomeric ATP synthase and subcomplexes would lead to higher mobility and redistribution away from cristae tips, similar as we recently observed in a different setup (Weissert et al. 2021). To assess this, we used Tracking and Localization Microscopy (TALM) (Appelhans and Busch 2017a) to map single ATP synthase molecules at nanometer resolution. We generated a stable hiPSC line expressing the F_1_ subunit γ (SUγ) fused C‐terminally to a Halo7 tag, enabling fluorescent labeling. Tagging of SUγ still enables F_O_ rotation (Prescott et al. 2003; Noji et al. 1997), however might affect rotational speed (Adachi 2012). Assembly of the SU γ‐Halo with ATP synthase in undifferentiated hiPSC was checked by BN‐PAGE and immunostaining, revealing bands corresponding to monomeric (V_M_), oligomeric (V_O_), and F_1_ subcomplexes, for example, F_1_‐c_8_ and F_1_ (He et al. 2018; Wittig, Carrozzo, et al. 2006; He 2020; Figure S7A). Interestingly, the ratio of subunit e/subunit β was lower in SUγ‐tagged hiPSC cardiomyocytes and further decreased in senescence conditions, suggesting weakening of dimers (Figure S2). Mitochondrial localization of labeled SUγ‐Halo was confirmed by its co‐localization with TMRE (Pearson coefficient: 0.558 ± 0.086), and CM differentiation was validated by α‐actinin staining and observation of spontaneous beating (Video S1 and Figure S7B,C).

**FIGURE 3:**
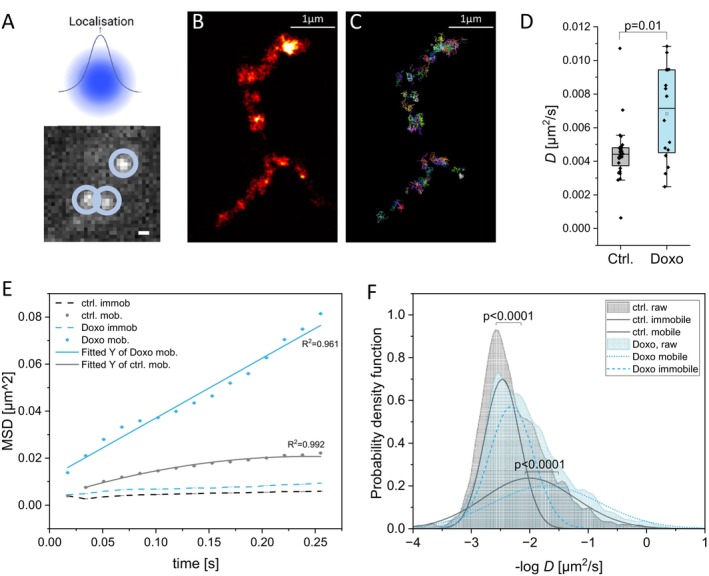
Single‐molecule localization and tracking of ATP synthase shows increased mobility of ATP synthase in senescent CM. Stably expressing SU γ‐Halo hiPSC‐CM were treated with 50 nM doxorubicin for 4 days (Doxo) or left untreated (Ctrl). (A) Exemplary single molecules as well as graphical illustration of the point spread function and localization. (B) Cumulative image of localizations from 5000 frames. False color: Hot. Every single dot is a single ATP synthase (C) Superimposed trajectories from 5000 frames. (D) Mean diffusion coefficient of ATP synthase; single dots represent the mean diffusion coefficient of one ATP synthase of one measurement including usually more than 100 trajectories. (E) Mean square displacement of ATP synthase in doxorubicin‐treated and control cells. Fitting merged two populations, a mobile (solid line) and an immobile population (dashed line). (F) Probability density function (PDF) of diffusion coefficients of single complexes (*n* = 16,796 trajectories for control and *n* = 3942 trajectories for senescent conditions). Mobile and immobile populations fitted with an amplified Gaussian fit. PDF fit of diffusion coefficients for immobile and mobile ATP synthase complexes. Statistical test: Mann–Whitney because of non‐normal distribution of the data. The vertical lines in the box blots represent the median values, the error bars denote the standard deviation (SD), the boxes represent the 25th to 75th percentile. Significance: **p ≤ 0.01. (B, C) Scale bar: 1 μm *n* = 4 biological replicates, 16,796 trajectories (Ctrl), 3942 trajectories (Doxo).

Using differentiated hiPSC‐CM, we performed live‐cell single‐molecule tracking. Sparse labeling enabled high‐resolution localization without signal overlap (Abdullah et al. 2019). Signals from individual ATP synthase molecules were fitted using 2D‐Gaussian models (Figure 3A), and videos of 1000–5000 frames yielded cumulative localization (Figure 3B) and trajectory maps (Figure 3C). The median diffusion coefficient (*D*) increased significantly in senescent cells (0.007 μm^2^/s vs. 0.004 μm^2^/s; *p* < 0.01; Figure 3D). Analysis of mean square displacement revealed that ATP synthase in senescent cells displayed free diffusion in a time range of 0.25 s, whereas control cells showed restricted diffusion (Figure 3E). Cumulative and probability density functions of median *D* values (Figure 3F–H) showed a clear shift toward higher mobility in senescent cells, affecting both mobile and quasi‐immobile ATP synthase populations. These findings indicate increased mobility and redistribution of ATP synthase in senescent cells, suggesting a spatiotemporal reorganization of ATP synthase along cristae membranes.

### 3.6 Lamellar Cristae in Senescent Mitochondria Display Fenestrations

**FIGURE 4:**
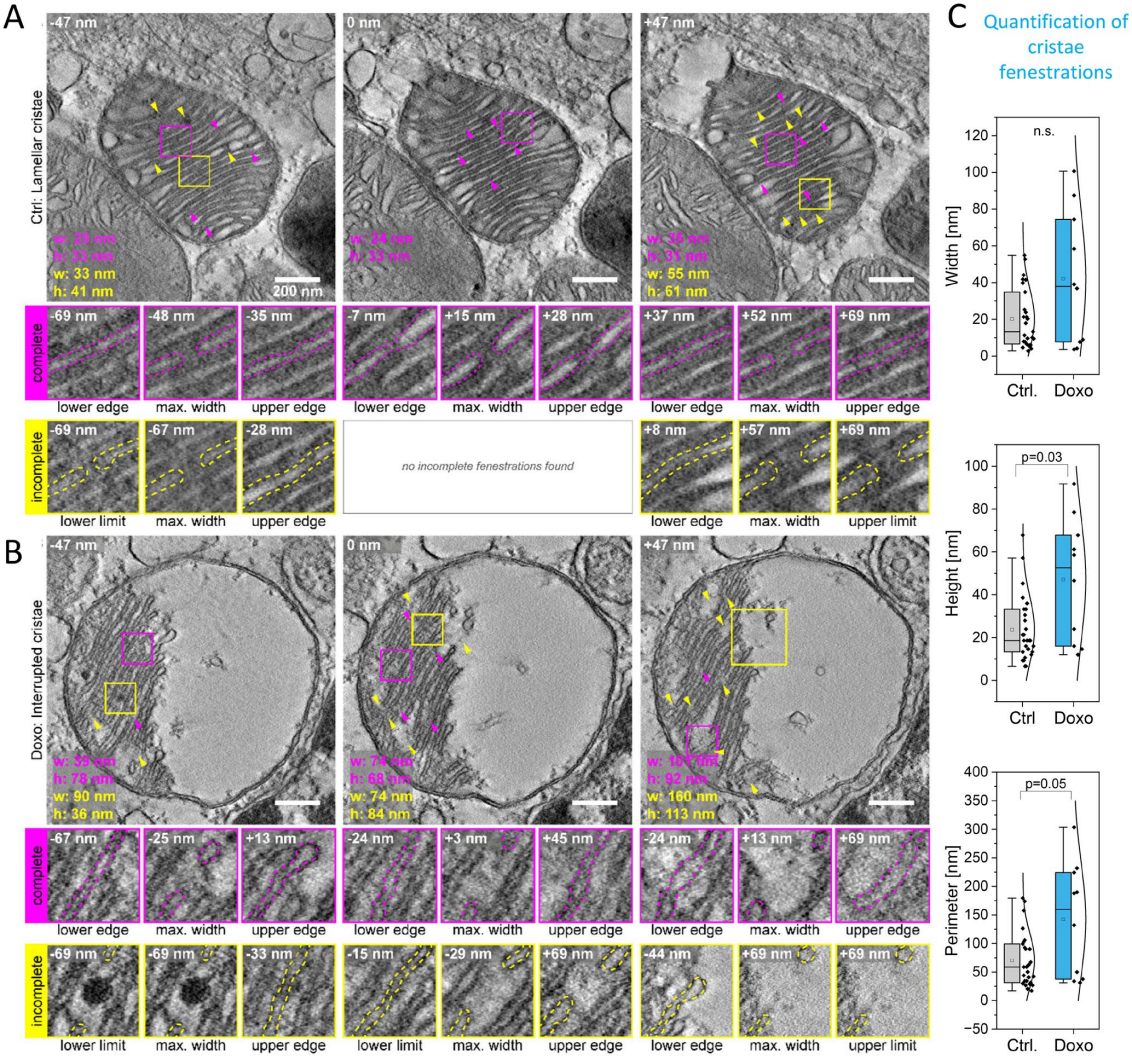
Senescence increases cristae fenestration size in rat NVCM. (A) Mitochondrion with lamellar cristae from control conditions (Ctrl). (B) Mitochondrion with interrupted cristae from senescent conditions (Doxo). Three axial planes of mitochondria, displayed 47 nm apart from each other, obtained by electron tomography. The white number in the upper left corner indicates the position of every image in the Z axis relative to the one in the center. Each image shows a detailed view of cristae, highlighting the appearance of discontinuities, which correspond to cristae pores, also known as fenestrations (arrowheads). Fenestrations with two edges were defined as complete (magenta), whereas fenestrations with an edge and a limit were defined as incomplete (yellow). An edge is the slice where the lamellae become continuous, whereas a limit is the last slice of the stack, where you can still see the discontinuity. For complete fenestrations, insets show the slices where you can see their lower edge, maximum width, and upper edge. For incomplete fenestrations, insets show the slices where you can see their lower limit/edge, maximum width, and upper edge/limit. The colored numbers written over the images represent the width (w) and height (h). The contrast of images was enhanced to improve visualization. (C) Quantification of cristae fenestrations according to Figure S9. Scale bar: 200 nm.

To explore whether the reorganization of ATP synthase and loss of SU e induces broader structural changes as reported before (Rabl et al. 2009), we analyzed cristae architecture in more detail by 3D electron tomography (3D‐ET). In our previous study, we used 2D TEM to show that doxorubicin‐treated NVCM exhibit a shift in mitochondrial morphology to larger, more circular forms, with a significant increase in disrupted cristae and matrix space devoid of cristae (lamellar: 68% vs. 48%; interrupted: 3% vs. 23%) (Morris et al. 2023; Figure S8). Here, we performed 3D‐ET on 200 nm sections of NCVM mitochondria. From reconstructed volumes (105 slices; 1277 × 1277 × 140 nm^3^, voxel size: 1.33 nm^3^; Videos S2 and S3), we selected mitochondria representative of each condition—lamellar cristae in controls and interrupted cristae in senescent cells. In Figure 4, we display three axial planes spaced 47 nm apart (Figure 4). Cristae appeared as flat lamellae spanning most of the stack. In controls, cristae extended across the entire mitochondrial width (Figure 4A), while in senescent cells, they did not reach the other side, resulting in an interrupted cristae phenotype (Figure 4B). Discontinuities within lamellae, visible in several adjacent slices (magenta arrowheads and insets), are known as cristae fenestrations (Adams 2023). Those represent “windows” that allow matrix mixing within mitochondria characterized by an intricated architecture, determined by packed cristae. The fenestrations were wider and taller in senescent mitochondria (quantification details in Figure S9) consistent with our previous observations in aged mouse cardiac tissue (Molina‐Riquelme et al. 2026). We also observed partial discontinuities (yellow arrowheads) that appeared only above or below certain slices (Figure 4, yellow insets), which we interpret as incomplete fenestrations that did not span the entire stack. In senescent condition, incomplete fenestrations were larger than in control cells (Figure 4B, yellow arrowheads). Also, a substantial space devoid of cristae, typically for aged mitochondria (Brandt et al. 2017), is present.

These data suggest that in neonatal cardiomyocytes under senescent conditions, the lateral continuity of mitochondrial cristae lamella is interrupted by fenestrations that show morphological adaptations.

### 3.7 Senescent Cardiomyocytes Exhibit Reduced Ca^2+^ Oscillation Frequency and Delayed Cytosolic Ca^2+^ Clearance

**FIGURE 5:**
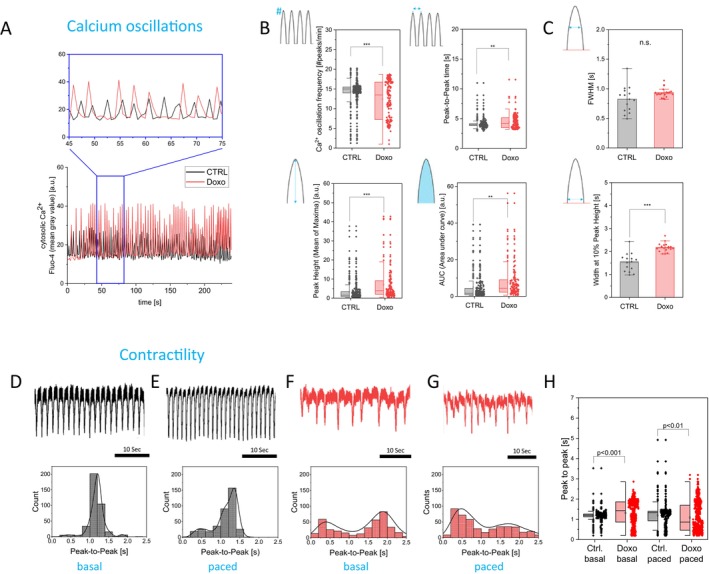
Senescent cardiomyocytes exhibit increased irregularity in Ca^2+^ oscillations and contractile activity. (A) Exemplary Fluo‐4 fluorescence intensity of single cells shows cytosolic Ca^2+^ oscillations in control and senescent CM. Calcium traces of beating CM were recorded over a period of 4 min with a frame rate of 0.74 s. A zoom is shown in the panel on top. Low pass filtering was used to analyze single traces. (B) Quantification of individual peak parameters (mean of all peaks per cell) shows a decreased peak frequency in Doxo‐treated CM compared to control. Additionally, an irregular Ca^2+^ oscillation pattern can be deduced from the high standard deviation in peak frequency and the decrease in Peak‐to‐Peak time. Furthermore, the peak amplitude is increased in senescent CM, as observed in the increased peak height and area under curve, *n*_ctrl_. = 285, *n*_doxo_ = 160 (technical replicates, single cells); *N* = 6 (biological replicates, videos). (C) Quantification of full peak width at 50% or 10% peak height reveals a tailing of peaks (extended decay phase of each transient) in Doxo‐treated CM, *n* = 18 (technical replicates, single cells); *N* = 3 (biological replicates, videos). Statistics for (C) and (D): One‐way ANOVA with Tukey post hoc test, the vertical lines in the box blots represent the median values, the error bars denote the standard deviation (SD), the boxes represent the 25th to 75th percentile. (D) An example trace of the contraction of non‐treated hiPSC‐CM is shown on the top. The histogram and the plotted Kernel‐Smooth‐Fit show a clear peak at 1.25 s peak‐to‐peak time. (E) Non‐treated CTRL hiPSC‐derived cardiomyocytes at 3 Hz pacing; an example trace is shown on the top. The histogram and the plotted Kernel‐Smooth‐Fit show a clear peak at 1.3 s peak‐to‐peak time. Furthermore, the fit is slightly shifted to the left compared to (D). (F) Example trace of contractility of doxorubicin‐treated hiPSC‐CM. The histogram and the plotted Kernel‐Smooth‐Fit show two peaks at 0.25 s and 1.75 s peak‐to‐peak time. (G) Doxorubicin‐treated hiPSC‐CM at 3 Hz pacing. An example trace is shown on the top. The histogram and the plotted Kernel‐Smooth‐Fit show two peaks at 0.40 s and 1.75 s peak‐to‐peak time. Statistics for (H): Mann–Whitney for paired measurement ctrl.‐doxo (basal; paced), the vertical lines in the box blots represent the median values, the error bars denote the standard deviation (SD), the boxes represent the 25th to 75th percentile. *n* = 2. Contractility measurements were done with the CardioExcyte 96 device (Nanion Technologies GmbH).

The contractile activity of CM critically depends on ATP availability and Ca^2+^ dynamics. In addition to driving contraction, ATP is required for Ca^2+^ clearance via PMCA and SERCA. We therefore hypothesized that the observed disorganization of ATP synthase and the associated ATP deficiency may alter Ca^2+^ oscillations in beating CM. To test this, cytosolic Ca^2+^ transients were visualized using the fluorescent indicator Fluo‐4‐AM (Hansen et al. 2017). For reference, cells were co‐stained with Fura‐Red. While Fluo‐4 reliably reported Ca^2+^ oscillations, Fura‐Red did not respond to Ca^2+^ fluctuations (Figure S10). Consequently, only Fluo‐4‐AM was used for subsequent analyses (Figure 5A).

Senescent CM exhibited a reduced oscillation frequency resulting in a prolonged peak‐to‐peak interval of Ca^2+^ transients (Figure 5B). Moreover, the area under the curve and peak amplitude were increased, accompanied by delayed Ca^2+^ clearance, evident as an extended decay phase of each transient in the lower 10% peak width of the individual peaks, but not at the full width at half maximum (Figure 5A,B upper panel). Collectively, this data indicates that ATP‐dependent cytosolic Ca^2+^ clearance from the cytosol is compromised under conditions of mitochondrial dysfunction.

### 3.8 Senescent Cardiomyocytes Exhibit Increased Irregularity in Contractile Activity

To test the physiological impact of disturbed calcium oscillations in conjunction with energy deficiency, we determined contractile activity of CM. We measured the contractility of control and senescent hiPSC‐CM with a CardioExcyte 96 device (Nanion Technologies GmbH), which allows for high temporal resolution. Traces of non‐treated hiPSC‐derived cardiomyocytes were analyzed. The histogram and the plotted Kernel‐Smooth‐Fit show a clear peak at 1.25 s peak‐to‐peak time (Figure 5C). Analysis of traces of contractility of doxorubicin‐treated hiPSC‐CM shows two peaks at 0.25 and 1.75 s peak‐to‐peak time. Next, control hiPSC‐CM were paced with 3 Hz. The histogram and the plotted Kernel‐Smooth‐Fit show a clear peak at 1.3 s peak‐to‐peak time (Figure 5C). Furthermore, the fit is slightly shifted to the left compared to non‐paced control CM. Finally, doxorubicin‐treated hiPSC‐CM were paced at 3 Hz. Analysis shows two peaks at 0.40 and 1.75 s peak‐to‐peak time (Figure 5D). The appearance of two peak‐to‐peak times in the senescent cells is a sign of contractile irregularity. The statistical analysis of all conditions also shows significant changes in mean peak‐to‐peak times for the paced control and senescent CM. Paced doxorubicin‐treated cells also show a significant difference in beating activity compared to non‐paced doxorubicin‐treated cells (Figure 5E). In sum, senescent CM displayed a more irregular beating behavior.

## 4 Discussion

In this study, we investigated the relationship between cristae structure, the spatiotemporal organization and function of ATP synthase, and Ca^2+^ dynamics in a hiPSC‐model of senescent heart cells. Primary‐aged human cardiomyocytes (CMs) for physiological studies are virtually inaccessible for physiological studies. However, human induced pluripotent stem cells (hiPSC) can be differentiated into functional, beating cardiomyocytes (hiPSC‐CMs) and treated with low‐dose doxorubicin to induce senescence (Karabicici et al. 2021; Wang et al. 2021; Marques et al. 2025). Like mouse and human embryonic stem cell‐derived cardiomyocytes, hiPSC‐derived CMs retain some fetal‐like properties (as the absence of T‐tubules (Gherghiceanu et al. 2011)) and exhibit considerable heterogeneity (Lundy et al. 2013; Yang et al. 2014; Lieu et al. 2009). However, culturing them as monolayers on Matrigel enhances homogeneity and calcium handling (Hwang 2015). As a result, hiPSC‐CMs are recognized as a suitable model for investigating cardiomyocyte pathophysiology (Garg 2018), aging and senescence (Emanet et al. 2025; Acun et al. 2019).

We recently developed a hiPSC‐CM–based senescence model that faithfully reproduces the senescent phenotype observed in neonatal ventricular cardiomyocytes (NVCMs) (Spallarossa et al. 2009; Maejima 2007). Senescence was confirmed using canonical markers, including γH2AX foci (Siddiqui et al. 2015) and enlarged nuclear area (Freund et al. 2012), as well as mitochondrial elongation (Figure S1 and previous work) (Mai et al. 2010). The expression of adult markers such as alpha‐actinin (Figure S8) and the maintenance of contractile function (Video S1) further validate this model as a robust platform for investigating senescent‐associated changes in mitochondrial ATP synthase dynamics at the cellular level. Whether cardiomyocyte‐specific T‐tubules exist in hiPSC‐CM is not finally clarified.

In our previous study, we reported reduced respiratory function and altered mitochondrial ultrastructure in senescent hiPSC‐CM. Most notably, we found a reduction of cristae density, reduction of matrix electron density and mitochondrial swelling and an increase in truncated cristae (Morris et al. 2023). In the present study, we show that the apparent cristae truncation can be partially assigned to fenestrations in lamellar cristae. This aligns well with electron tomography (ET) analysis of aged human and mouse heart tissue from our laboratories revealing that these apparent truncations actually represent increased cristae fenestrations in aged cardiomyocytes (Molina‐Riquelme et al. 2026). Such fenestrations in cristae of cardiomyocytes have been described before (Adams 2023), but the observation that they change in senescence is new. We suggest that the destabilization of ATP synthase dimers and oligomers in combination with re‐localization of mobile ATP synthase contributes to the observed alterations in cristae architecture. Oligomeric ATP synthase is known to play a critical role in shaping cristae morphology (Paumard et al. 2002; Rabl et al. 2009; Blum et al. 2019), and its disruption could directly contribute to the re‐structuring of cristae observed in senescent cardiomyocytes.

Cristae are the sides of oxidative phosphorylation (Gilkerson et al. 2003) and their re‐structuring could affect ATP production. Indeed, we found reduced mitochondrial ATP levels in senescent cardiomyocytes. This was also accompanied by an increased capacity for ATP hydrolysis by mitochondrial ATP synthase, which can contribute to reduced mitochondrial ATP levels (Acin‐Perez et al. 2023). Additionally, the increased frequency of mPTP opening reduces ΔΨ_m_ and, consequently, the proton motive force for ATP synthesis. Consistent with this, we previously reported reduced oxygen consumption rates linked to ATP production in the same cell model (Morris et al. 2023).

To investigate the underlying mechanisms, we asked how changes in ATP synthase organization might be linked to ATP hydrolysis and mPTP opening. We found that the weakening of ATP synthase dimers and oligomers was associated with increased ATP synthase mobility in senescent cardiomyocytes resulting in re‐localization of ATP synthase into regions of lower PMF (Rieger et al. 2021; Weissert et al. 2021; Appelhans and Busch 2017b). Lower PMF is a trigger for reverse ATP synthase activity and ATP hydrolysis.

The mobility of ATP synthase is influenced by its oligomerization status, membrane architecture and physicochemical properties—including lipid composition (Appelhans 2012; Ramadurai et al. 2010). Fenestrations in lamellar cristae restrict lateral protein diffusion, artificially reducing apparent mobility and leading to an underestimation of the diffusion coefficient (*D*). In senescent cells, enlarged fenestrations further exacerbate this underestimation. Accurate determination of *D* therefore requires 3D tracking. Additonally, age‐related changes in the lipid composition of the inner mitochondrial membrane, as reported in aging heart tissue (Pepe et al. 1999; Paradies 1999; Lewin and Timiras 1984; Lee 2006), may alter membrane viscosity and further impact ATP synthase mobility.

To record ATP synthase mobility, we used a fluorescently labeled SU‐γ‐HaloTag, which preferentially labeled monomers and subcomplexes (Figure S7B). This observation was similar to what we previously reported with pHluorin‐tagged SU γ (Rieger et al. 2021). Subcomplexes, as described by He et al. (2018), are likely composed of F_1_‐c_8_ and F_1_, respectively. The increased mobility of ATP synthase in SU γ‐tagged senescent hiPSC‐cardiomyocytes indicates that more ATP synthase dimers were broken. This may be related to the decreased amount of SU e in senescent SU γ‐tagged cells (Figure S2A).

In sum, we suggest that these factors—dimer and monomer destabilization, cristae remodeling, and lipid composition—together contribute to the altered spatiotemporal organization of ATP synthase in senescent cardiomyocytes, which changes the localization of ATP synthase in cristae. Clustering of ATP synthase at cristae rims is thought to be important for efficient ATP production (Weissert et al. 2021; Gerle 2020, 2024; Davies 2011); therefore, displacement of ATP synthase from cristae tips into regions with decreased proton motive force (Strauss et al. 2008; Rosselin et al. 2018; Marx and Busch 2024) likely impairs ATP synthesis and favors ATP hydrolysis. Consistently, aging has been associated with a loss of ATP synthase dimers (Bou‐Teen et al. 2022; Daum et al. 2013) and elevated ATP hydrolysis. The increased mobility and relocalization of ATP synthase thus provide a mechanistic explanation for these age‐related energetic deficits.

**FIGURE 6:**
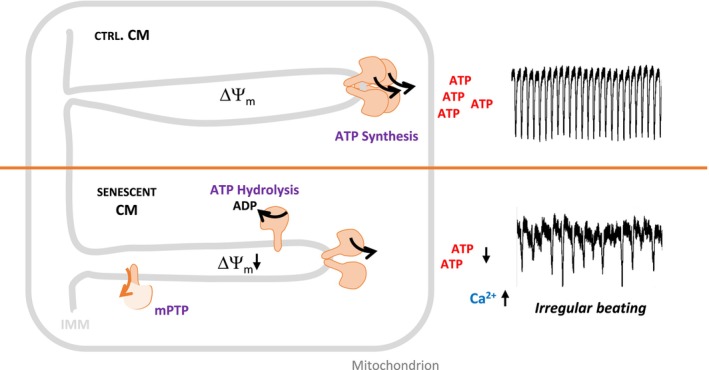
Graphical abstract. Relation between altered spatiotemporal organization and function of ATP synthase, impaired Ca^2+^‐dynamics, and contractile dysfunction in senescent cardiomyocytes.

In addition, the increased SU c/SU β ratio indicates alterations in the stoichiometry of F_O_ and F_1_ components of ATP synthase. An increase of F_O_‐c‐subunit levels (Alavian 2014), raises the propensity for mitochondrial permeability transition pore (mPTP) formation (Gerle 2020). This is consistent with our observation of increased mitochondrial membrane potential (ΔΨ_m_) flickering and enhanced cobalt ion accessibility to mitochondria, both indicative of mPTP opening (Figure 2). Increased mPTP opening is a known feature of aged mouse hearts (Bou‐Teen et al. 2022) and has been proposed as an adaptive mechanism to facilitate Ca^2+^ exchange between mitochondria and the cytosol in senescent cells (Protasoni et al. 2024). Cyclophilin D (CypD), a known stimulator of mPTP, mediates its opening in senescent cells and is vital for their survival (Protasoni et al. 2024). High levels of CypD also weaken ATP synthase dimer formation (Beutner et al. 2017). The increased transient opening of the mPTP likely functions as pressure valve for elevated cytosolic Ca^2+^ in cardiomyocytes (Lu 2016). Based on our observations, we suggest that reduced mitochondrial ATP levels compromise ATP‐dependent Ca^2+^ extrusion mechanisms at the plasma membrane and sarcoplasmic reticulum, leading to elevated Ca^2+^ spikes and delayed recovery between transients (Figure 5). This results in increased cytosolic Ca^2+^ pressure, which, together with CypD, likely enhances mitochondrial Ca^2+^ uptake and further stimulates mPTP opening (Popoiu et al. 2023). Increased mPTP opening leads to decreased ΔΨ_m_, reducing the efficiency of ATP production and potentially promoting reverse ATPase function to maintain the ΔΨ_m_ (Figure 6). Related to this, mitochondrial PMF is lowered during cytosolic Ca^2+^ elevation (Poburko et al. 2011) as a consequence of mitochondrial Ca^2+^ uptake, which translates into mitochondrial calcium transients and stimulation of matrix dehydrogenases by Ca^2+^, followed by ETC‐activation and determining the voltage‐dependence activity of mitochondrial ATP synthase (Wescott et al. 2019). Mutually, these changes result in cytosolic ATP and Ca^2+^ dysregulation, manifesting as irregular beating patterns in paced senescent cardiomyocytes (Gorski et al. 2015).

Together, our findings indicate that enhanced ATP hydrolysis, increased ΔΨ_m_ flickering, and destabilization of ATP synthase dimers are associated with reduced ATP availability in senescent CM. This energy deficiency may contribute to disturbed Ca^2+^ oscillatory dynamics and impairment of contractile function observed in senescent cardiomyocytes, involving altered ATP synthase stoichiometry and spatiotemporal reorganization within altered cristae, which imposes unfavorable structural and functional constraints for ATP synthesis.

## Author Contributions

S.M., N.M., I.M.‐R., S.P., G.S., and V.E. and K.B.B. designed the experiments, validated the data. S.M., N.M., I.M.‐R., G.B., F.S., S.P., and K.B.B. performed the experiments. S.M., G.S., V.E., and G.B. contributed to the methodology. K.B.B., G.S., H.E.V., and V.E. provided resources; S.M., V.E., and K.B.B. wrote the manuscript; K.B.B., S.M., N.M., G.B., and S.P. visualized the study; all authors supervised the data; K.B.B. and V.E. contributed to project administration; K.B.B., H.E.V., and V.E. acquired funding. All authors have read and agreed to the published version of the manuscript.

## Funding

The study was supported by a grant from the BMFTR/DLR (FKZ 01DN19046) for S.M./K.B., and PCI/ANID‐BBMFTR (180060) for V.E. and H.E.V. and K.B. Also, FONDECYT (1191770 and 1231557) for V.E., and ANID Ph.D. fellowship 21201041 for I.M.‐R.

## Conflicts of Interest

The authors declare no conflicts of interest.

## Supporting information


**Data S1:** Supporting information S1.

**Data S2:** Supporting information S1.

**Data S3:** Supporting information S1.

**Data S4:** Supporting information S1.

## Data Availability

Raw data of single molecule tracking will be made available upon request.
