# Supplementary material for: Disruption of ATP Synthase Spatiotemporal Organization, Ca2+ Dynamics, and Contractile Function in Senescent Cardiomyocytes
Source: Aging Cell. 2026 Jan 28;25(2):e70388. doi: 10.1111/acel.70388 (PMC12848789; doi:10.1111/acel.70388)
Supplement: Supplementary file 1 — Data S1: Supporting information S1. [file ACEL-25-e70388-s001.pdf]

Supplementary Information

Supplementary Methods

Mitochondria swelling assay

Swelling of isolated mitochondria was evaluated by measuring the absorbance at 540 nm. The optical density (OD) was measured in 7 s intervals using a Cytation 5 (Agilent) plate reader in the kinetic readout mode. Briefly, 25 µg of freshly isolated HeLa mitochondria were resuspended in 125 mM KCl, 2mM K_2HPO_4, 1mM $MgCl_2$, 20 mM HEPES, pH 7.4 supplemented with 5 mM succinate and 2 µM rotenone. PTP opening was triggered by the addition of 100 µM $CaCl_2$. At the end of the experiment, 0.1% saponin was added to measure maximal mitochondrial swelling. The fraction of swollen mitochondria was calculated as described [1, 2].

Supplementary Figures

Figure S 1: Senescence markers are increased in doxorubicin-treated cardiomyocytes. Increase in DNA double strand breaks, nuclear area and mitochondrial elongation in senescent cells. (A) Immunestaining of double strand breaks (dsb) indicated by phosphorylated yH2A.X. H2AX is a variant of the histone H2A protein. When a double-strand break occurs, H2AX is rapidly phosphorylated at serine 139, forming γ-H2AX. DAPI was used to visualize the nucleus. (B) Quantification of cell nuclei exhibiting yH2A.X signals. (C) Area of nuclei in control and doxorubicin treated cells was determined from z-projections of DAPI signals using the “analysis of particles” plugin in ImageJ. Scale bars: 20 µm. For (B) and (C), n=2 independent experiments. For dsb analysis, N=109 (control) and N=113 (doxorubicin) nuclei were analyzed. For nuclei area analysis, N=45 (control) and N=44 (doxorubicin) nuclei were analyzed. (D) Mitochondrial morphology in ctrl. and senescent hiPSC-CM. TMRE staining (10 nM), n=2. (E) Quantification of mitochondrial morphology in areas of 10 x 10 μm^2. Statistics: Mann-Whitney test used for (B)(C)(E).

Figure S 2: Organization of ATP synthase in hiPSC-CM and NCVM. (A) Relative changes of ATP synthase subunit SU e/ SU β and SU e / SU c and SU c / SU β expression in control and doxorubicin-treated hiPSC-CM; n=5 independent treatments. (B) BN-PAGE was conducted for separation of oligomers (O, including dimers) and monomers (M) of ATP synthase in control and senescent NCVM. The relative amount of O, M and subcomplexes was determined and the ratio of M/O calculated. n=3 independent preparations, two technical replicates (not shown).

Figure S 3: TMRE fluorescence measurements to indicate $\Delta\Psi_m$. (A) TMRE responds to the uncoupler FCCP as indicated. (B) TMRE fluorescence bleaching over a time period of 43 seconds, which is typically used to determine flickering events.

Figure S 4: Co^{2+}/Calcein quenching assay to determine leakiness of mitochondria. (A) Scheme showing the staining procedure to determine mitochondrial leakiness: Mitotracker™DeepRed (MTDR) is used to stain mitochondria (and serves as a mask to identify mitochondria); calcein-AM, which enters both the cytoplasm and mitochondria; and Co^{2+}, which enters the cytosol and quenches cytosolic calcein. When mitochondria are leaky, due to opening of the mitochondrial permeability transition pore (mPTP) or after induction of mPTP via ionomycin, Co^{2+} also enters mitochondria and quenches mitochondrial calcein. (B) From left to right: images showing staining of mitochondria with MTDR, cellular calcein fluorescence, quenching of cytosolic calcein by Co^{2+} and quenching of mitochondrial calcein due to leaky mitochondrial membranes.

Figure S 5: Doxorubicin treatment does not increase the mitochondrial swelling rate in isolated HeLa mitochondria under these experimental conditions. Mitochondrial swelling assay of freshly

isolated mitochondria from HeLa cells shows no direct effects of doxorubicin treatment on mPTP opening. (A) Stimulation with 100 µM calcium chloride induces mitochondrial swelling of freshly isolated energized mitochondria. (B) Blocking mPTP opening by treatment with 10 µM CsA decreases the basal mitochondrial swelling rate. (C) Swelling assay of isolated mitochondria treated with control or doxorubicin (50 nM) displayed as fraction of swollen mitochondria. (D) Doxorubicin treatment does not increase the mitochondrial swelling rate in isolated mitochondria.

Figure S 6: Apoptosis is not enhanced in senescent CM. Immune-staining of caspase 3 as readout for apoptosis. Pro-caspase 3 is expected to appear at ~35 kDa and the cleaved active caspase 3 at 15-17 kDa.

Figure S 7: Fluorescence-tagging of ATP synthase in hiPSC-CM via HaloTag-fusion and labeling. (A) Vector KA0637-pPgCAG-rtTAM2-IN to generated stable integration of SU γ-HaloTag via PiggyBac system. This vector contained a G418 resistance gene for selection of positive clones, as well as the rTA transactivator sequence that allows doxycycline-inducible expression of SU γ-HaLo. (B) Analysis of ATP synthase organization in an undifferentiated hiPSC wild-type cell line and a cell line that stably expresses the subunit γ- with HaloTag at its C-terminus using Blue-Native PAGE and immunostaining with α–SU γ and α-HaloTag. V_O: oligomeric, V_M: monomeric ATP synthase, F1-c8 and F1: subcomplexes. Two preparations are shown. (C) Successful differentiation of hiPSC-SU γ-HaloTag expressing cells into cardiomyocytes indicated by CM marker α-actinin. (D) Mitochondrial localization of subunit γ-HaloTag stained with JF646.

Figure S 8: Mitochondrial and cristae ultrastructure is altered in senescent NVCM. (A) Representative electron micrographs of the mitochondrial network in Ctrl and Doxo NVCM. (B) Representative images of single mitochondria with lamellar and interrupted cristae, for Ctrl and Doxo NVCM, respectively. Scale bars: 1 µm (A), 300 nm (B).

Figure S 9: Cristae fenestration analysis by electron tomography. (A) To perform electron tomography, single-tilt image series were acquired by TEM (i), followed by tomogram alignment to generate a reconstructed volume (ii), from which we selected a ROI containing the mitochondrion of interest (iii). (B) Unbiased analysis for cristae fenestrations. The total number of slices of the ROI was counted (i), to divide it into equal sub-ROIs (ii), from which the central slice, colored in cyan, was selected (iii). In each of the central slices, every complete and incomplete fenestration was identified (iv). Finally, one complete and one incomplete fenestration from every central slice were randomly selected and further characterized (v). For a better understanding of the workflow, the proportion of the Z axis is augmented. (C) Example of cristae fenestration analysis for the upper central slice of the mitochondrion from the control condition. All the complete and incomplete fenestrations were identified, and one of each was randomly selected (boxes). For the complete fenestration, the slice where the width of the discontinuity became maximum (middle inset), and the slices where the lamella became continuous (upper and lower insets) were selected. For the incomplete fenestration, the slice where the width of the discontinuity became maximum (middle inset), the slice where the ROI ended (upper inset), and the slice where the lamella became continuous (lower inset) were selected. Cartoons on the bottom show the lamella observed from the front to portray the slices selected in each step. (D) Morphometric analysis of cristae fenestrations. The widths were quantified by measuring the distance of the discontinuity in XY (left), whereas the heights were quantified by measuring the distance of the discontinuity in Z (middle). To better describe the parameters of complete and incomplete fenestrations, the terms “maximum” and “partial” were used, respectively. For complete fenestrations, we assumed they resembled two half-ellipses, since their width and height were not the same, and did not perfectly intersect in the middle. This way, the perimeter and area were estimated using equations based on regular ellipses (left).

Figure S 10: Ca^{2+} dynamics in beating control and senescent hiPS-CM. (A) Overlay of Fluo-4 and Fura-red stained beating hiPS-CM with (left) and without (right) transmission image. (B) Fluo-4 and Fura-red signals in beating CM over a time period of 20 s.

Supplementary videos

Supplementary Video 1. Ca^{2+} dynamics in beating control and senescent hiPS-CM. (A) Overlay of Fluo-4 and Fura-red stained beating hiPS-CM with (left) and without (right) transmission image. (B) Fluo-4 and Fura-red signals in beating CM over a time period of 20 s.

Supplementary Video 2. Tomographic volume of a mitochondrion with lamellar cristae from a control NVCM. The video displays a volume of 1277×1277×140 nm^3, with 105 slices, and a voxel size of 1.33×1.33×1.33 nm^3.

Supplementary Video 3. Tomographic volume of a mitochondrion with interrupted cristae from a doxorubicin-treated NVCM. The video displays a volume of 1277×1277×140 nm^3, with 105 slices, and a voxel size of 1.33×1.33×1.33 nm^3.

References

1. Carraro, M., et al., *Molecular nature and regulation of the mitochondrial permeability transition pore(s), drug target(s) in cardioprotection.* J Mol Cell Cardiol, 2020. **144**: p. 76-86.
2. Petronilli, V., et al., *Physiological effectors modify voltage sensing by the cyclosporin A-sensitive permeability transition pore of mitochondria.* J Biol Chem, 1993. **268**(29): p. 21939-45.

Figure S 1

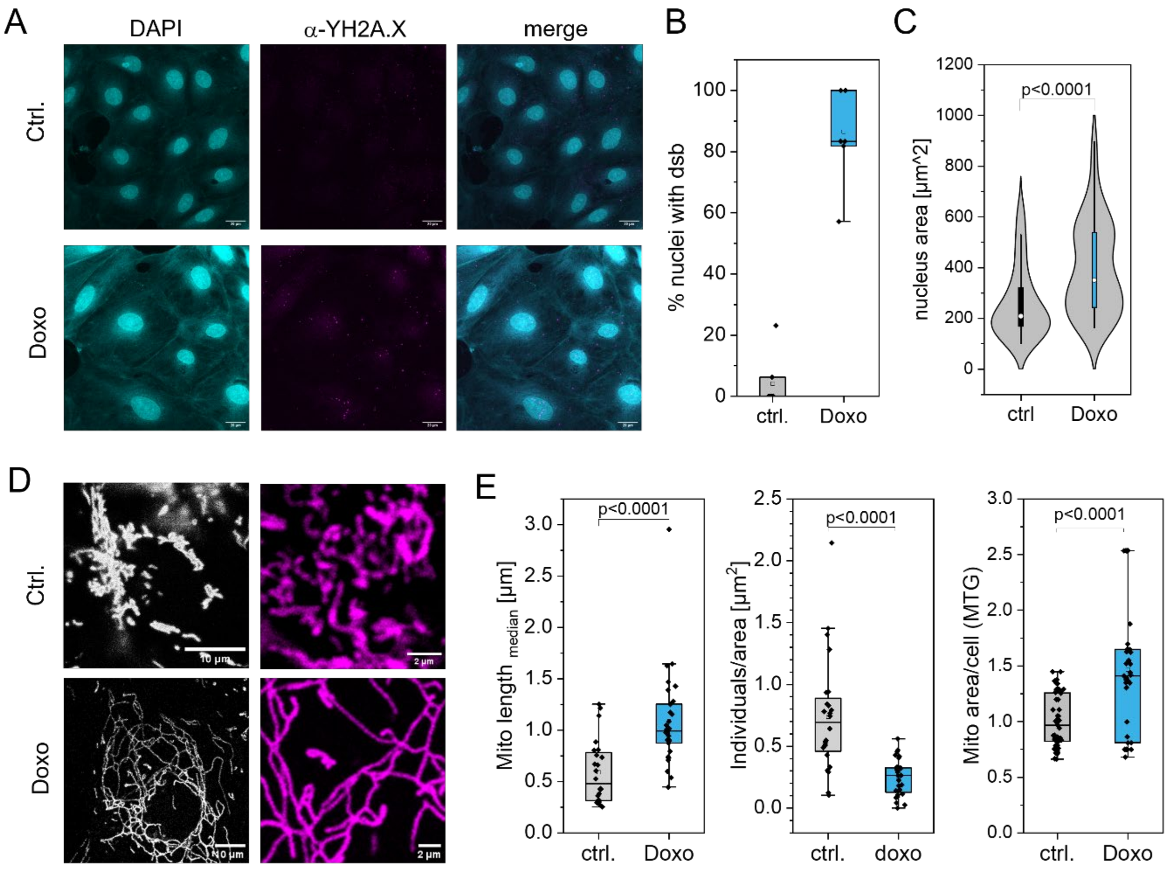
A
DAPI
α-YH2A.X
merge
Ctrl.
Doxo
B
% nuclei with dsb
0
20
40
60
80
100
ctrl.
Doxo
C
nucleus area [µm^2]
0
200
400
600
800
1000
1200
p<0.0001
ctrl
Doxo
D
Ctrl.
Doxo
10 µm
2 µm
E
Mito length median [µm]
0.0
0.5
1.0
1.5
2.0
2.5
3.0
p<0.0001
ctrl.
Doxo
Individuals/area [µm2]
0.0
0.5
1.0
1.5
2.0
2.5
p<0.0001
ctrl.
doxo
Mito area/cell (MTG)
0.0
0.5
1.0
1.5
2.0
2.5
3.0
p<0.0001
ctrl.
Doxo

Figure S 2

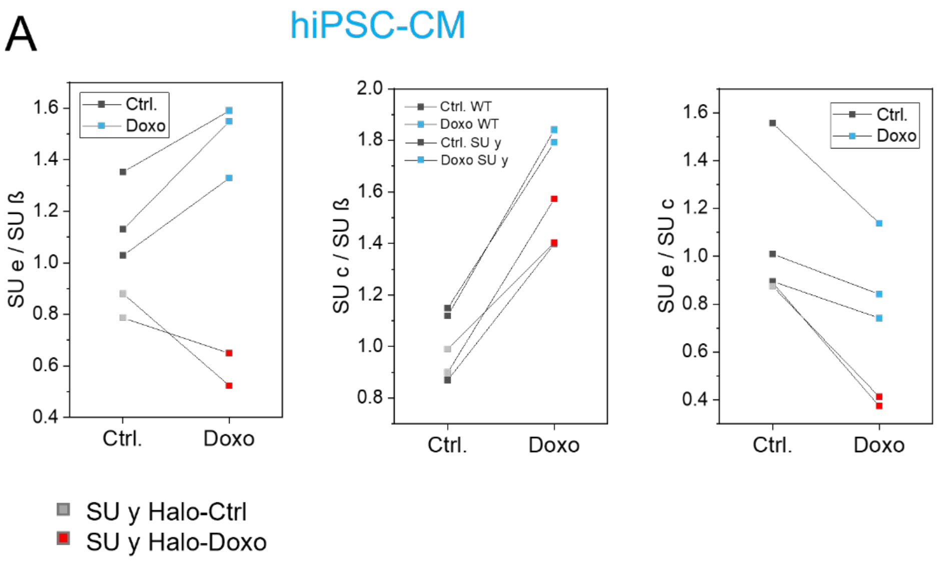
A
hiPSC-CM
SU e / SU ß
SU c / SU ß
SU e / SU c
Ctrl.
Doxo
Ctrl. WT
Doxo WT
Ctrl. SU y
Doxo SU y
SU y Halo-Ctrl
SU y Halo-Doxo

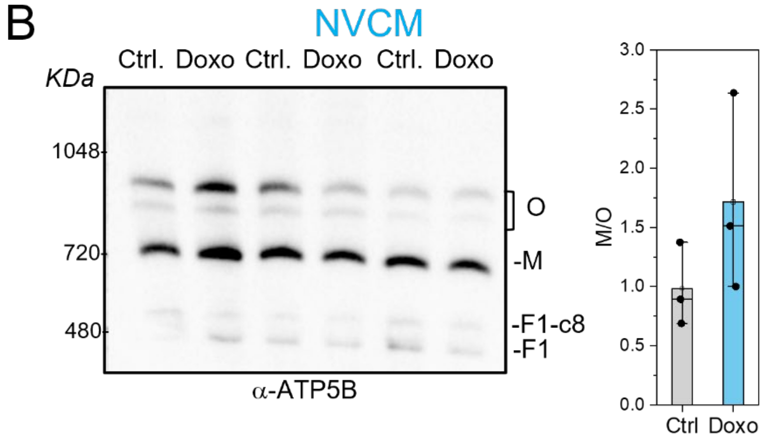
B
NVCM
Ctrl. Doxo Ctrl. Doxo Ctrl. Doxo
KDa
1048
720
480
O
-M
-F1-c8
-F1
α-ATP5B
M/O
Ctrl
Doxo

Figure S 3

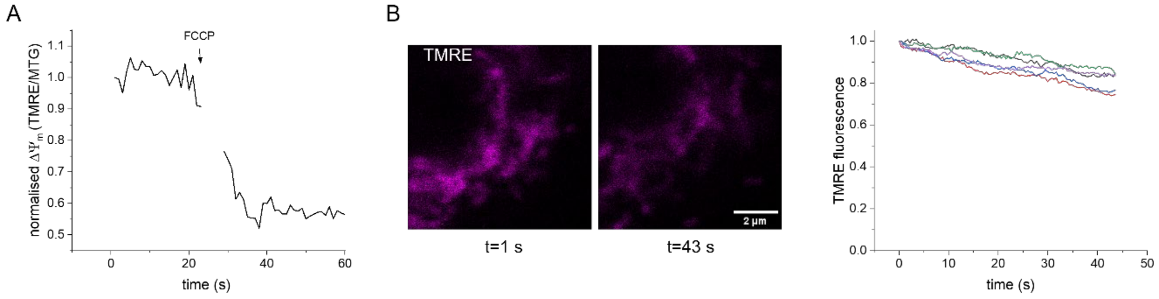
A
FCCP
normalised ΔΨm (TMRE/MTG)
time (s)
B
TMRE
2 µm
t=1 s
t=43 s
TMRE fluorescence
time (s)

Figure S 4

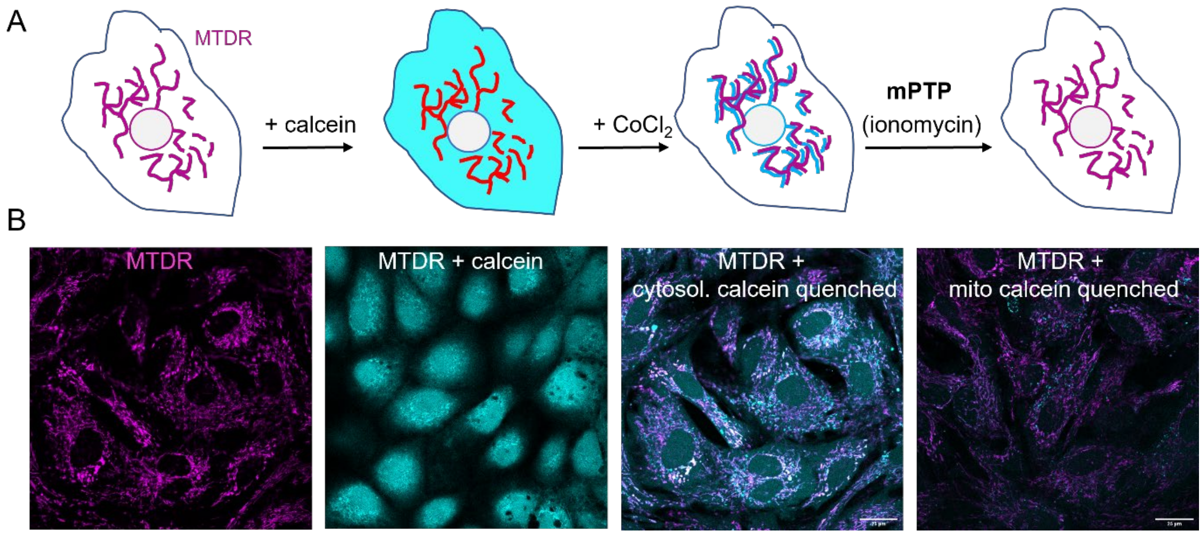
A
MTDR
+ calcein
+ CoCl2
mPTP
(ionomycin)
B
MTDR
MTDR + calcein
MTDR +
cytosol. calcein quenched
MTDR +
mito calcein quenched

Figure S 5

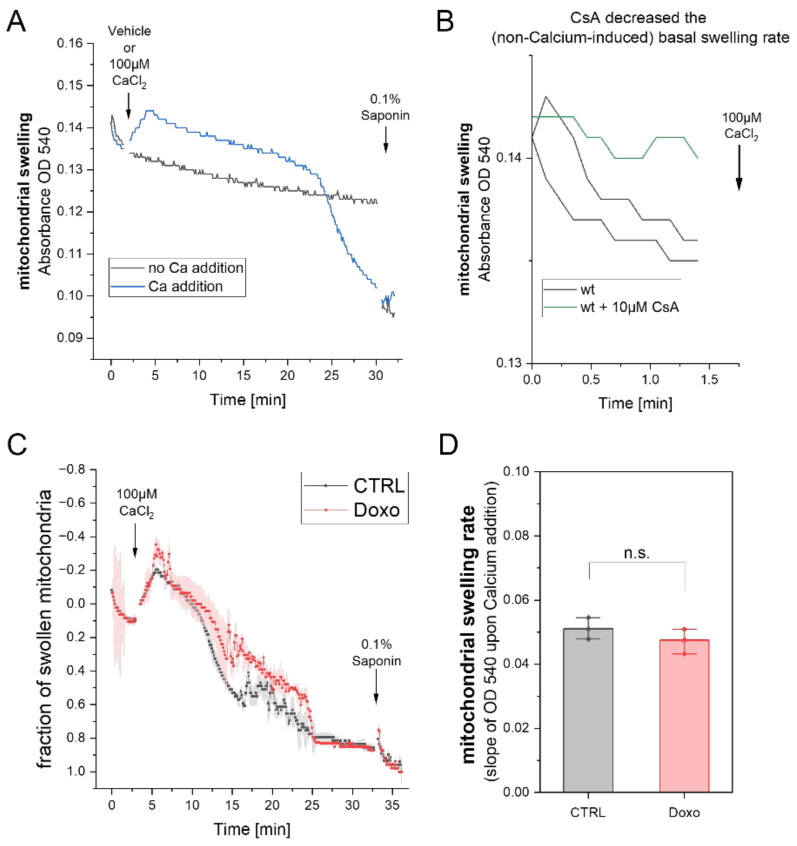
A
Vehicle or 100µM CaCl2
0.1% Saponin
mitochondrial swelling
Absorbance OD 540
no Ca addition
Ca addition
Time [min]
B
CsA decreased the (non-Calcium-induced) basal swelling rate
100µM CaCl2
wt
wt + 10µM CsA
Time [min]
C
100µM CaCl2
0.1% Saponin
CTRL
Doxo
fraction of swollen mitochondria
Time [min]
D
mitochondrial swelling rate
(slope of OD 540 upon Calcium addition)
n.s.
CTRL
Doxo

Figure S 6

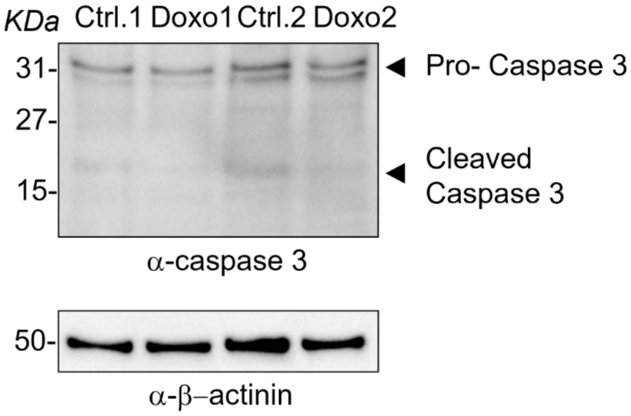

KDa
Ctrl.1 Doxo1 Ctrl.2 Doxo2
31-
27-
15-
Pro- Caspase 3
Cleaved Caspase 3
α-caspase 3
50-
α-β–actinin

Figure S 7

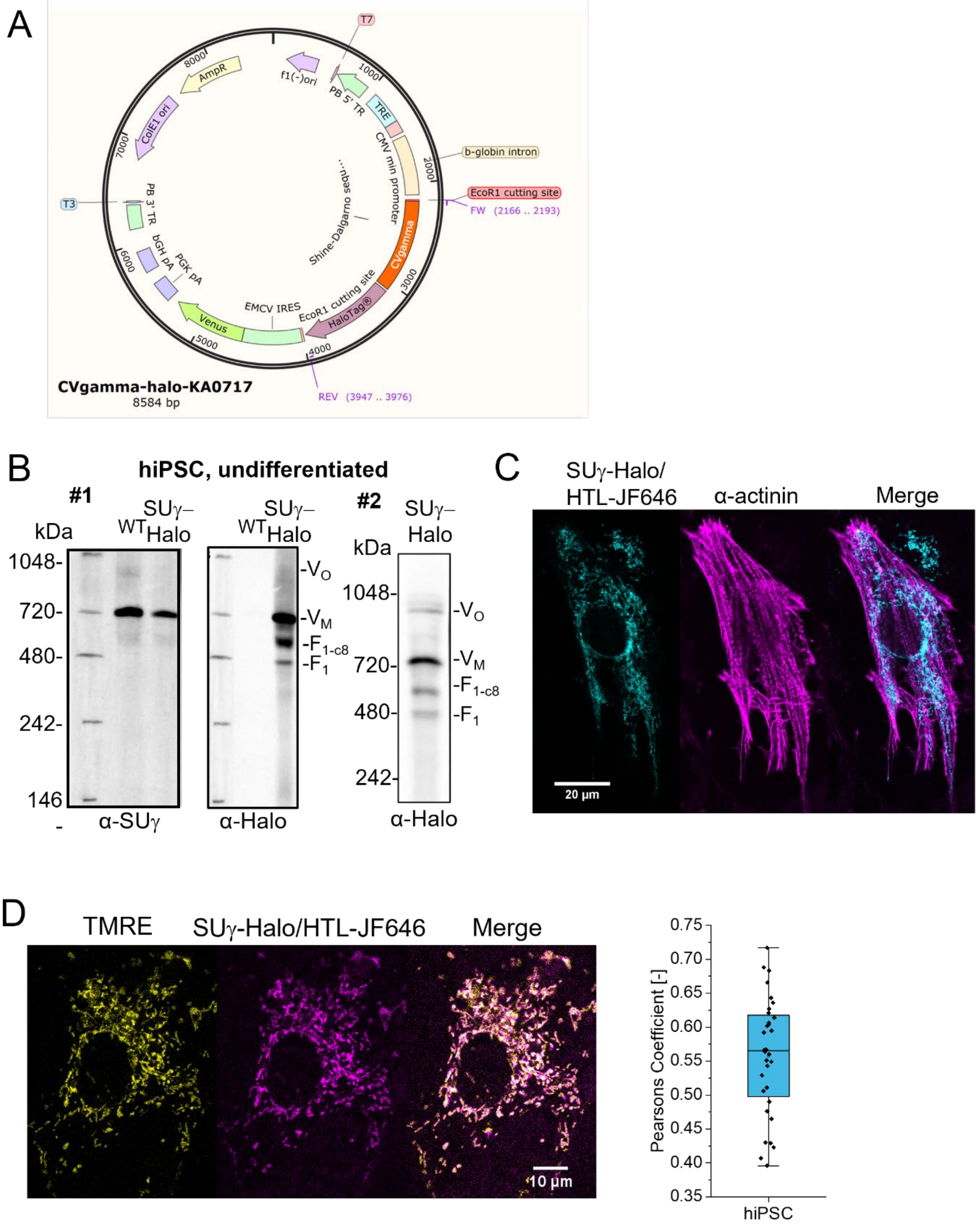
A
T7
f1(-)ori
1000
PB 5' TR
TRE
CMV min promoter
b-globin intron
2000
EcoR1 cutting site
FW (2166 .. 2193)
CVgamma
3000
HaloTag®
EcoR1 cutting site
EMCV IRES
4000
REV (3947 .. 3976)
Venus
5000
PGK pA
bGH pA
6000
PB 3' TR
T3
7000
ColE1 ori
AmpR
8000
Shine-Dalgarno sequ...
CVgamma-halo-KA0717
8584 bp
B
hiPSC, undifferentiated
#1
kDa
WT
SUγ–Halo
1048-
720-
480-
242-
146
-V_O
-V_M
-F_1-c8
-F_1
α-SUγ
α-Halo
#2
SUγ–Halo
kDa
1048-
720-
480-
242-
-V_O
-V_M
-F_1-c8
-F_1
α-Halo
C
SUγ-Halo/
HTL-JF646
α-actinin
Merge
20 µm
D
TMRE
SUγ-Halo/HTL-JF646
Merge
10 µm
Pearsons Coefficient [-]
0.75
0.70
0.65
0.60
0.55
0.50
0.45
0.40
0.35
hiPSC

Figure S 8

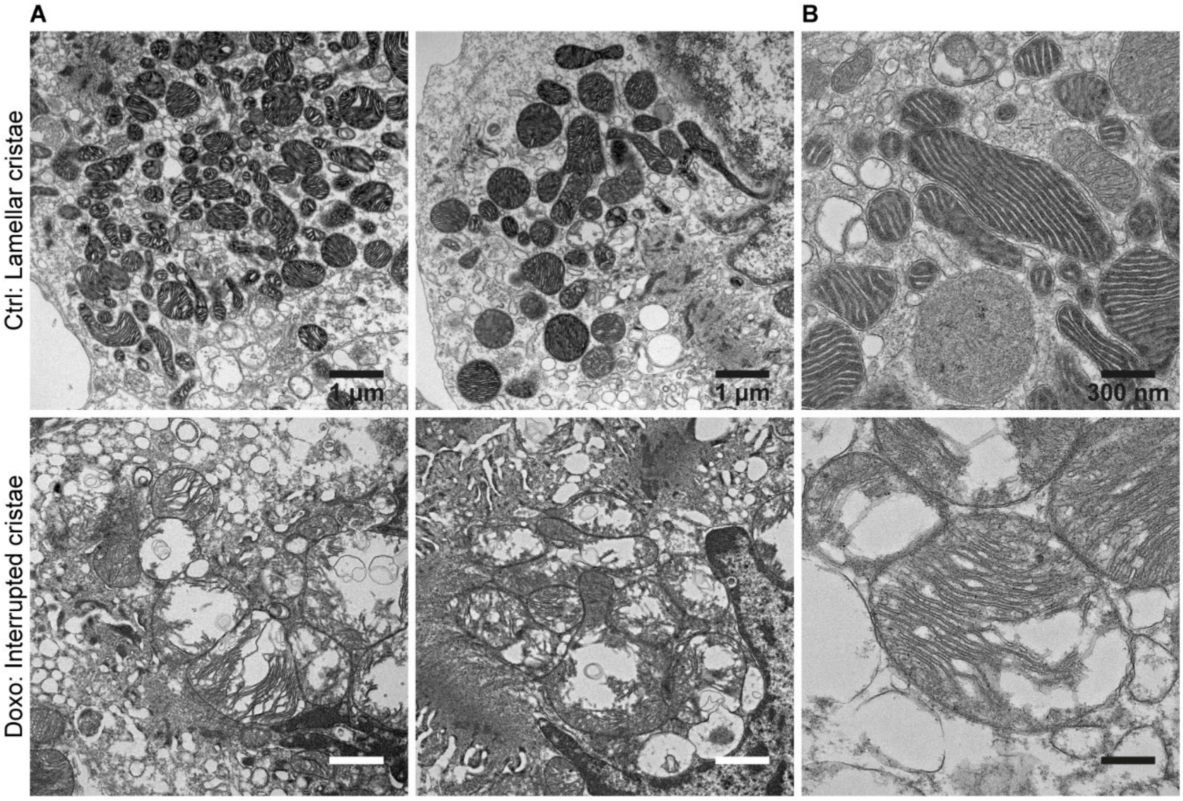
A
B
Ctrl: Lamellar cristae
Doxo: Interrupted cristae
1 µm
1 µm
300 nm

Figure S 9

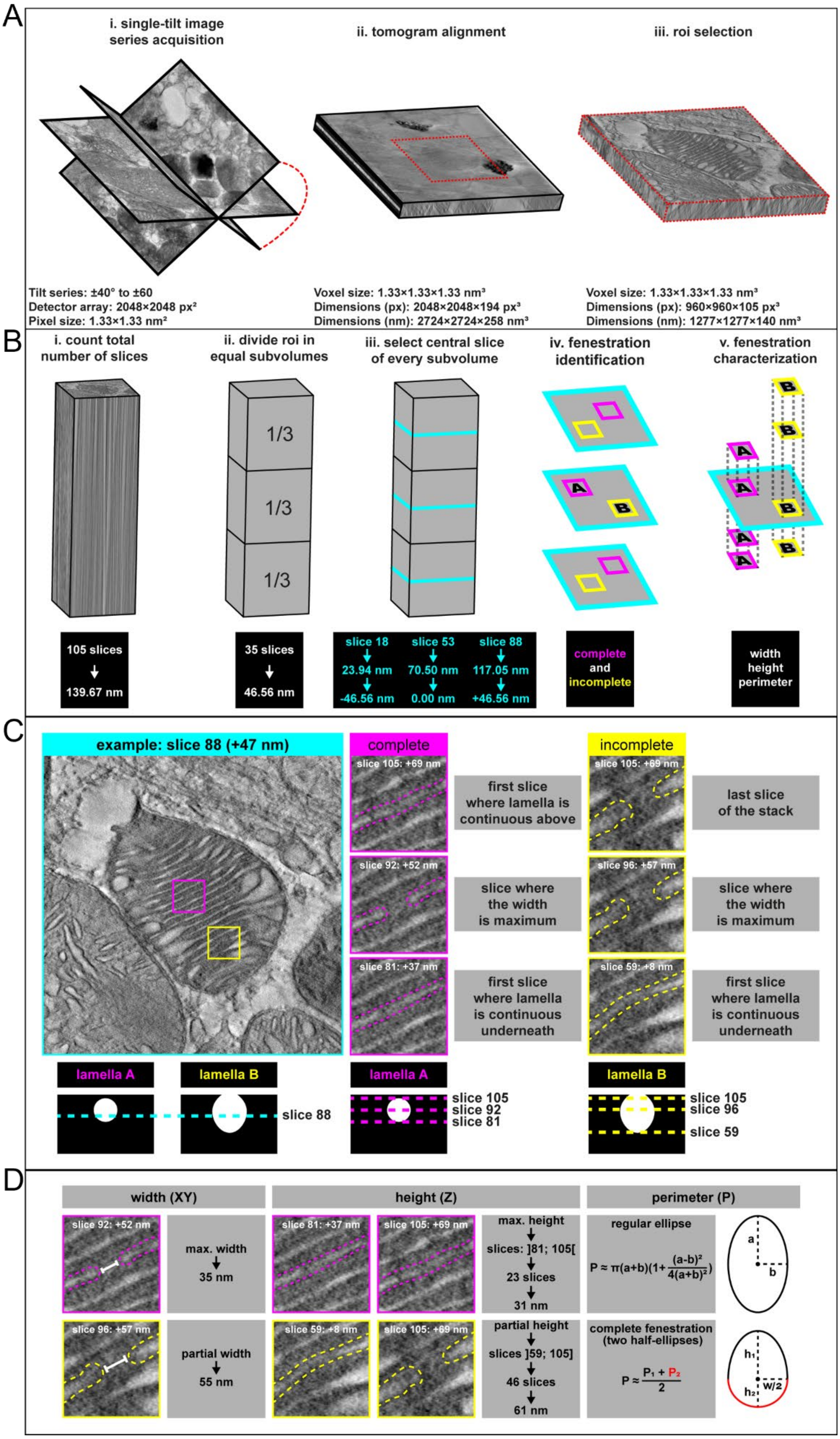
A
i. single-tilt image series acquisition
ii. tomogram alignment
iii. roi selection
Tilt series: ±40° to ±60
Detector array: 2048×2048 px²
Pixel size: 1.33×1.33 nm²
Voxel size: 1.33×1.33×1.33 nm³
Dimensions (px): 2048×2048×194 px³
Dimensions (nm): 2724×2724×258 nm³
Voxel size: 1.33×1.33×1.33 nm³
Dimensions (px): 960×960×105 px³
Dimensions (nm): 1277×1277×140 nm³
B
i. count total number of slices
ii. divide roi in equal subvolumes
iii. select central slice of every subvolume
iv. fenestration identification
v. fenestration characterization
1/3
1/3
1/3
105 slices
139.67 nm
35 slices
46.56 nm
slice 18
23.94 nm
-46.56 nm
slice 53
70.50 nm
0.00 nm
slice 88
117.05 nm
+46.56 nm
complete and incomplete
width height perimeter
C
example: slice 88 (+47 nm)
complete
slice 105: +69 nm
first slice where lamella is continuous above
slice 92: +52 nm
slice where the width is maximum
slice 81: +37 nm
first slice where lamella is continuous underneath
incomplete
slice 105: +69 nm
last slice of the stack
slice 96: +57 nm
slice where the width is maximum
slice 59: +8 nm
first slice where lamella is continuous underneath
lamella A
lamella B
slice 88
lamella A
slice 105
slice 92
slice 81
lamella B
slice 105
slice 96
slice 59
D
width (XY)
height (Z)
perimeter (P)
slice 92: +52 nm
max. width
35 nm
slice 81: +37 nm
slice 105: +69 nm
max. height
slices:]81; 105[
23 slices
31 nm
regular ellipse
P ≈ π(a+b)(1+(a-b)²/4(a+b)²)
slice 96: +57 nm
partial width
55 nm
slice 59: +8 nm
slice 105: +69 nm
partial height
slices]59; 105]
46 slices
61 nm
complete fenestration (two half-ellipses)
P ≈ (P1 + P2)/2
h1
h2
w/2

Figure S 10

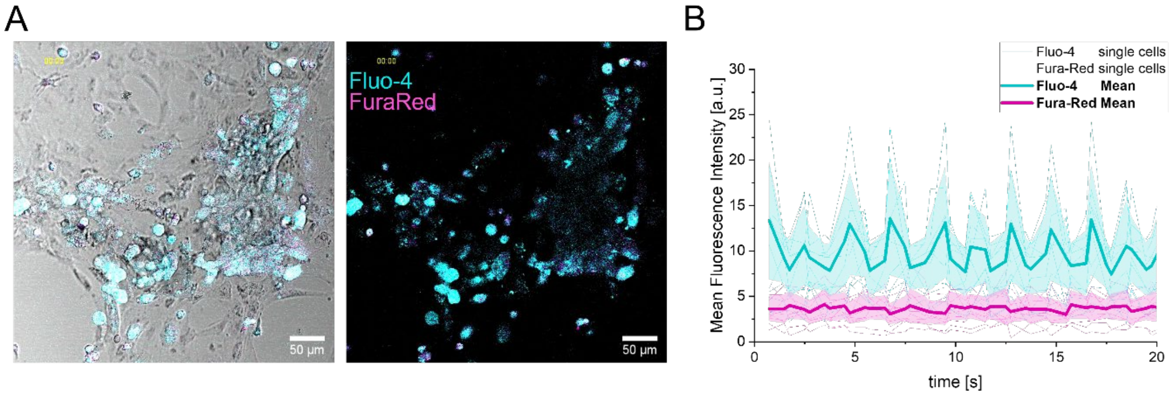
A
Fluo-4
FuraRed
50 µm
50 µm
B
Fluo-4 single cells
Fura-Red single cells
Fluo-4 Mean
Fura-Red Mean
Mean Fluorescence Intensity [a.u.]
time [s]
0
5
10
15
20
25
30
